# METTL3 mediated m6A modification of HKDC1 promotes renal injury and inflammation in lead nephropathy

**DOI:** 10.7150/ijbs.112463

**Published:** 2025-05-31

**Authors:** Xiao-guo Suo, Jia-nan Wang, Qi Zhu, Meng-meng Zhang, Qing-lin Ge, Li-jin Peng, Yue-yue Wang, Ming-lu Ji, Yang-mei Ou, Ju-tao Yu, Hao Lu, Xin-ran Cheng, Bing-bing Hou, Xin Chen, Sai Zhu, Xiang-yu Li, Chao Li, Shuai-shuai Xie, Chen Yang, Feng-he Li, Juan Jin, Fang Wang, Xiao-ming Meng

**Affiliations:** 1 Inflammation and Immune Mediated Diseases Laboratory of Anhui Province, Anhui Institute of Innovative Drugs, School of Pharmacy, Anhui Medical University, Hefei, 230032, China; 2 Department of Urology, The First Affiliated Hospital of Anhui Medical University, Hefei 230032, China; 3 Institute of Nephrology, Affiliated Hospital of Guangdong Medical University, 57 Renmin Road, Zhanjiang 524001, China; 4 Department of Pharmacy, Lu'an Hospital of Anhui Medical University, Lu'an People's Hospital of Anhui Province, Lu'an, 237006, China

**Keywords:** METTL3, Lead nephropathy, HKDC1, Renal inflammation

## Abstract

Environmental and industrial Pb exposure poses a significant public health challenge. Acute exposure to high Pb concentrations can result in renal injury. Here, we revealed that N6-methyladenosine (m6A) RNA methylation was significantly upregulated in lead nephropathy and was mainly mediated by the methyltransferase METTL3. Functionally, METTL3 knockout in renal tubular epithelial cells or AAV9-mediated METTL3 silencing alleviated renal injury and the inflammatory response induced by lead acetate. METTL3 silencing in renal tubular epithelial cells reduced both m6A RNA methylation and inflammatory responses following lead acetate treatment. We identified hexokinase domain-containing 1 (HKDC1), known to function in the glycolytic pathway, as a direct METTL3 target. Importantly, HKDC1 was upregulated at both mRNA and protein levels after lead acetate treatment, thereby promoting renal injury and inflammation. Mechanistically, HKDC1 binds to ATPB and antagonizes the ubiquitinase Murf1, thereby leading to increased expression of ATPB and activation of the NF-κB signaling pathway, which promotes renal inflammation. We further confirmed that STM2457, an inhibitor of METTL3, protected against renal injury and inflammation induced by lead acetate. Collectively, our study demonstrated that the METTL3/HKDC1 axis is a potential target for the treatment of lead nephropathy, and STM2457 is expected to be a protective agent against renal injury caused by lead acetate.

## Introduction

Lead nephropathy is a kidney disease caused by Pb poisoning. This is mainly due to long-term occupational exposure to Pb-containing compounds, through occupations such as mining, smelting, and battery manufacturing, or lead pollution in the living environment, such as the long-term consumption of alcohol, beverages, and food stored in lead containers 1-3. As of 2019, there are a total of 5-13 million Pb poisoning patients in China 4. Approximately 15% of these patients develop Pb-induced kidney disease. The main pathological features include renal tubular injury, renal interstitial fibrosis, renal vascular disease, and renal atrophy 5. The pathogenesis of lead nephropathy has not been fully elucidated, and there is an urgent need for specific preventive and therapeutic drugs in clinical practice. Therefore, exploring its pathogenesis and treatment methods has become an important topic in recent years both domestically and internationally.

Epigenetic modifications play an important role in the occurrence and development of various diseases, and their role in kidney diseases is being increasingly studied 6. Research has shown that epigenetic modifications regulate the function and dialogue between intrinsic and non-intrinsic renal cells by altering DNA methylation, histone modifications, and non-coding RNA (ncRNAs) without altering the nucleic acid sequence, thereby affecting the occurrence and development of kidney disease 7. N6 methyladenosine (m6A) is one of the most common and abundant RNA modifications 8. Increasing evidence suggests that m6A RNA modification is a dynamic and reversible event. The synergistic effect of methyltransferases (m6A writers) and demethylases (m6A erasers) facilitates the deposition and consumption of this modification. METTL3, METTL14, and WTAP have been shown to act as m6A methyltransferases in mammalian cells 9. Among these, METTL3 serves as the catalytic core, METTL14 serves as the structural support for METTL3, and WTAP stabilises the core system. The m6A erasers composed of FTO and ALKBH5 act as demethylases to reverse m6A modification 10,11. At the molecular level, m6A can regulate the splicing, translation, and decay rates of mRNA, thereby affecting protein production and various biological processes, such as cell differentiation, embryonic development, and stress response 12,13. Currently, research on m6A methylation is mainly focused on the nervous system, embryonic development, and tumour-related diseases, and its role in the kidneys has begun to receive attention. Our team focused on the study of epigenetics and kidney diseases. We previously reported that the m6A methylase METTL3 participates in acute kidney injury (AKI) via regulating the m6A methylation of TAB3 14. Simultaneously, we also found that METTL3 increases the modification of EVL m6A and regulates renal fibrosis through an IGF2BP2 dependent manner and proposed that the traditional Chinese medicine monomer Forsythia suspensa glycoside may be an inhibitor of METTL3 to alleviate chronic kidney disease 15. In clinical practice, the number of patients with kidney damage caused by Pb heavy metals in the environment is increasing; however, key issues such as its pathogenesis, prevention and treatment targets, and treatment methods still need to be addressed.

Preliminary experiments showed that the intraperitoneal injection of lead acetate administered over two weeks resulted in the most significant kidney damage in mice. We constructed *in vivo* and *in vitro* models of lead acetate-induced kidney injury and found that the levels of m6A methylation and expression of the m6A methylase METTL3 were significantly increased. To evaluate the influence of METTL3 -dominated m6A modifications, we performed relevant experimental studies using Ksp-Cre-METTL3 (Flox/Flox) conditional knockout (cKO) mice and small interfering RNA (siRNA)-silenced renal tubular epithelial cells. By performing RNA sequencing (RNA-seq) and methylation RNA immunoprecipitation sequencing (MeRIP-seq) on the tubular regions of mouse kidney tissues, we investigated the potential targets of METTL3-mediated m6A modifications and the detailed mechanisms of action. Subsequently, we investigated the function and possible mechanism of action of HKDC1 in lead nephropathy. Importantly, we evaluated the efficacy of METTL3-targeted therapy for lead nephropathy. These results provide a theoretical basis for the discovery of new targets for the prevention and treatment of lead nephropathy, which has important clinical significance and scientific research value.

## Materials and Methods

### Cell culture, transfection, and treatment

Human kidney tubular epithelial cells (HK2) were cultured in 5% fetal bovine serum (FBS)-containing DMEM/F12 medium at 95% air and 5% CO_2_ incubator (Thermo Fisher, USA). After overnight starvation in DMEM/F12 medium with 0.5% FBS, HK2 cells were treated with a lead acetate solution. The HK2 cells were transfected with METTL3 or HKDC1 siRNA or METTL3 or HKDC1 plasmids using lipo3000 according to the manufacturer's instructions and then stimulated with lead acetate at a concentration of 5 mM or control buffer (vehicle) for 24 h. To detect the therapeutic effect of STM2457, The HK2 cells were pretreated with different concentrations of STM2457 (2.5 μM, 5 μM, 10 μM) for 12 h, and then stimulated with lead acetate for 24 h for subsequent detection.

### MTT assay

HK2 cells were seeded in 96-well plates, and 100 μL of cell suspension was added to each well. At 70% - 80% confluence, the cells were treated with different concentrations of lead acetate (0.313, 0.625, 1.25, 2.5, 5, or 10 mM) for 24 h. Then, 10 μL of 3-(4,5-dimethylthiazol-2-yl)-2,5-diphenyl tetrazolium bromide (MTT, 5 mg/mL, Sigma-Aldrich Corporation) was added to each well, and the cells were incubated at 37 °C for 4 h. The medium was carefully discarded, 150 μL of DMSO was added to dissolve the formazan crystals, and the absorbance was measured at 492 nm using a microplate reader (Bio-Tek, Vermont, USA).

### RNA m6A dot blot assay

An RNA m6A dot blot assay was used to measure the m6A content in the poly (A) tailing of total RNA. Briefly, after total RNA was extracted from HK2 cells and kidney tissues, denatured at 95 ºC for 5 min and quickly cooled. The mRNA was loaded onto two nylon membranes (Sigma-Aldrich, GERPN1210B). After crosslinking under ultraviolet (UV) light for 60 min, one of the membranes as loading control was dyed with 0.02% methylene blue (Sangon Biotechnology, China) and the spot mark was observed. Next, the membrane was blocked with 5% skimmed milk prepared with buffer (TBST), and then the membrane was incubated with anti-m6A antibody (Abcam, Cambridge, UK) at 4 ºC overnight. After incubation with the secondary antibody, the membranes were visualised using an imaging system.

### Quantification of the m6A modification

Total RNA was isolated using Trizol reagent (Invitrogen, 15596018) according to the manufacturer's instructions and treated with deoxyribonuclease I (Sigma-Aldrich). RNA quality was analysed using a NanoDrop spectrophotometre. Changes in global m6A mRNA levels were measured using the EpiQuik m6A RNA Methylation Quantification Kit (Epigentek) following the manufacturer's protocol.

### mRNA stability assay

To analyse mRNA stability, the transfected HK2 cells were treated with transcription inhibitor actinomycin D (5 µg/mL). The HK2 cells were then collected and total RNA was extracted using Trizol reagent at different time points (0, 3, and 6 h after actinomycin D treatment). Reverse transcription was performed, and mRNA levels were measured by RT-qPCR. The mRNA levels were normalised to the expression level at 0 h.

### RNA immunoprecipitation

RNA immunoprecipitation (RIP) was conducted using the Magna RIP RNA-Binding Protein Immunoprecipitation Kit 607 (Millipore) according to the manufacturer's instructions. Briefly, approximately 1×10^7^ HK2 cells were cross-linked with 0.3% formaldehyde to strengthen the combination of RNA and proteins. Magnetic beads were mixed with 5 µg of Anti-m6A (Abcam) or anti-rabbit IgG (Abcam), and the treated magnetic beads were added to HK2 cell lysates, and the mixtures were incubated at room temperature for 4 h. Thus, a magnetic bead-antibody-protein-RNA complex was formed. Next, protease K digestion buffer was added to digest the complex at 55 ºC for 45 min. RNA was extracted using a phenol: chloroform: isoamyl alcohol buffer (125:24:1). The extracted RNA was analysed using Real-time PCR. IgG was used as a negative control to exclude non-specific binding.

### MeRIP-qPCR

The protocol for methylated RNA immuneprecipitation (MeRIP) was previously described 14. Further enrichment was calculated by qPCR, and the corresponding m6A enrichment in each sample was calculated by normalisation to the input.

### M6A-MeRIP-Seq

MeRIP-seq was performed as previously described. Briefly, total RNA was isolated using Trizol reagent (Invitrogen), and mRNA was enriched using the Dynabeads mRNA Purification Kit (Invitrogen). Next, RNA Fragmentation Reagents (AM8740, Invitrogen) were used to shear the RNA into approximately 100-nt fragments. Approximately 1/10 of the fragmented RNA was used as an input control for RNA sequencing. The remaining fragmented RNA was mixed with 50 μL of Dynabeads Protein A (Life Technology) pre-mixed with 16 μg of anti-m6A antibody at 4 °C overnight in IP buffer (150 mM NaCl, 10 mM Tris-HCL, and 0.1% NP-40 supplemented with RNase inhibitor and protein inhibitor). The bead-antibody-RNA mix was washed twice with a high-salt washing buffer, twice with a middle-salt washing buffer, and twice with a low-salt washing buffer. Following the last wash, 500 μL of Trizol was added to the mix to extract the binding RNA. Both the input and m6A IP samples were prepared for next-generation sequencing (NGS) using RiboBio (China).

### RNA-Seq

RNA-seq, high-throughput sequencing, and data analysis were performed using LC-BIO (Hangzhou, China). Total RNA was extracted from renal tissues using the Trizol Reagent (Invitrogen, cat. No 15596026), and DNA digestion was carried out after RNA extraction using DNase I. Qualified RNAs was quantified by Qubit3.0 with the QubitTM RNA Broad Range Assay kit (Life Technologies, Q10210). 2 μg total RNAs were used for stranded RNA sequencing library preparation using KCTM Stranded mRNA Library Prep Kit for Illumina® following the manufacturer's instruction. PCR products corresponding to 200-500 bp were enriched, quantified, and sequenced using a DNBSEQ-T7 sequencer (MGI Tech Co., Ltd. China) using a PE150 model. Raw sequencing data were first filtered using Trimmomatic (version 0.36), low-quality reads were discarded and the reads contaminated with adaptor sequences were trimmed, and clean data were mapped to the reference genome of mice using STRA software (version 2.5.3a) with default parameters. Reads mapped to the exon regions of each gene were counted using feature counts (Subread-1.5.1; Bioconductor) and RPKMs were calculated. Differentially expressed genes between groups were identified using the edgeR package (version 3.12.1). A p-value cutoff of 0.05 and a fold-change cutoff of 2 were used to judge the statistical significance of gene expression differences. Gene ontology (GO) analysis and Kyoto Encyclopedia of Genes and Genomes (KEGG) enrichment analysis for differentially expressed genes were both implemented using KOBAS software (version 2.1.1) with a p-value cutoff of 0.05 to judge statistically significant enrichment. Alternative splicing events were detected by using rMATS (version 3.2.5) with an FDR value cutoff of 0.05 and an absolute value of Δψ of 0.05.

### Mice

Mice were provided by the Experimental Animal Centre of Anhui Medical University (Hefei, China). All procedures involving experimental animals were performed following protocols approved by the Animal Experimentation Ethics Committee of Anhui Medical University and conformed to the Guide for the Care and Use of Laboratory Animals. Ethics committee approval number LLSC20221086. C57BL/6J male mice aged 8-10 weeks (body weights 20-23 g) were used. All animals were maintained under constant humidity and temperature at standard facilities under specific pathogen-free conditions, with free access to water. After anesthesia with Telazol, the experimental animals were humanely euthanized according to the Animal Experimentation Ethics Committee of Anhui Medical University.

### Renal tubular epithelial cell-specific METTL3 knockout mice

To specifically knockout METTL3 in renal tubular epithelial cells, we generated a mouse line by crossing females of the METTL3-floxed line (METTL3^fl/fl^) with the Ksp-Cre (B6.Cg-Tg (Ksp1.3-cre) 91Igr/J, male, aged 8-9 weeks) transgenic strain obtained from Shanghai Model Organisms Center, Inc. Filial 1 progeny, mice (male or female), and litters with heterozygous deletion of the METTL3 gene (METTL3^fl/+^) that harboured the Ksp1.3/Cre transgene (METTL3^fl/+^; Ksp1.3-Cre) were obtained and further crossed with the opposite sex of METTL3^fl/fl^ mice to obtain mice expressing a complete deletion of METTL3 in filial 2 progeny (METTL3^fl/fl^; Ksp1.3-Cre), referred to as METTL3.

### Adeno-associated virus infected mice

To knockdown METTL3/HKDC1 in the kidney, Adeno-associated virus (AAV9)-mediated delivery was employed. C57BL/6J mice (male, 8-10 weeks, Experimental Animal Centre, Anhui Medical University, Hefei, China) were used. AAV9 encoding mouse METTL3 and HKDC1 were provided by Hanheng Biotechnology (Shanghai, China). Briefly, the tails of mice were wiped with alcohol to expand the tail vein for injection. Mice were slowly injected with 100 μL lentivirus with a concentration of 1.5 × 10^12^ vg/mL through tail vein using a 0.5 mL insulin syringe at the speed of approximately 0.2 μL/min. Four weeks after lentiviral infection, subsequent model construction was initiated.

### Assessment of Renal Function

Blood samples collected from mice with or without an intraperitoneal injection of lead acetate after 14 days were used to measure creatinine and blood urea nitrogen (BUN) levels. The levels of creatinine and BUN in the blood samples were measured using a Creatinine and BUN Assay Kit (Nanjing Jiancheng, China) according to the manufacturer's instructions.

### Renal histology

To evaluate histological damage, periodic acid-Schiff (PAS) staining was performed according to the manufacturer's instructions. Briefly, the mouse kidneys were immediately fixed in 4% paraformaldehyde. After dehydration, the samples were embedded in paraffin. The paraffin sections were de-paraffinised and hydrated in a graded ethanol series. According to the manufacturer's instruction, PAS staining was performed in paraffin sections (4 µm) to assess the degree of tubulointerstitial damage and examined by light microscope. The proximal renal impairment score shows the extent of tubular necrosis and dilatation as follows: 0 = normal, 1 = 10%, 2 = 11% - 25%, 3 = 26% - 50%, 4 = 51% - 75%, 5 = 76% - 95%, and 6 = more than 96%. Images were captured using a BX51 microscope (Olympus, Center Valley, PA, USA). Quantification was performed by three investigators who were blinded to the experimental conditions.

### Immunohistochemical staining

Immunohistochemical assays were performed as described previously 16. The mouse kidneys were fixed in 4% paraformaldehyde for at least 24 h and embedded in paraffin. The paraffin sections were de-paraffinised and hydrated in a graded ethanol series. Antigens were retrieved by boiling the sections in a sodium citrate antigen repair buffer for 20 min. Endogenous peroxidase activity was blocked by incubation in 3% hydrogen peroxide. The sections were incubated overnight at 4 ºC with primary antibodies. The sections were then incubated with corresponding secondary antibodies. 3,3'-diaminobenzidine (DAB) was used as the chromogen. Sections were lightly counterstained with haematoxylin, dehydrated, and covered with coverslips. The images were acquired using an Olympus microscope.

### Immunofluorescence staining

Immunofluorescence analysis was performed as previously described 16. For kidney tissues immunofluorescence staining, the sections were repaired by microwave heating antigen with EDTA antigen repair solution (PH 9.0), and blocked with 10% BSA for 30 min at room temperature. Then, sections were incubated at 4 ºC overnight with the primary antibodies. Fluorescently labelled secondary antibodies were used. The slides were counterstained with DAPI. Samples were analysed, and pictures were taken using an Olympus microscope. For immunofluorescence staining, cells were fixed on a cover glass with paraformaldehyde and sealed with 10% BSA at room temperature for 30 min. Then, the sections were incubated with the primary antibody at 4 ºC overnight. After 90 min of incubation with CY3 or FITC, the cells were counterstained with DAPI for 10 min. Finally, an anti-fluorescence quencher was used to seal the sections and photographs were taken using an Olympus microscope.

### Western blotting

Western blot was performed as previously described 16. Briefly, Western blot was performed to detect protein expression in the renal tissues or cells. Renal tissues and cells were lysed in lysis buffer (RIPA:PMSF=1:100). The whole lysates were heated (100 ºC) for 10 min with 5×SDS-PAGE loading buffer (Beyotime). Proteins were then separated by polyacrylamide gel electrophoresis (80V, 30 min and 120V, 60 min) in acrylamide gels (10%) and transferred using a Bio-Rad western system (128V, 60 min) to NC membranes, which were immediately placed in 5% non-fat milk for blocking (1.5 h at room temperature). Membranes were then washed in TBST buffer for 5 min, followed by incubation with specific primary antibodies at 4 ºC overnight. The membranes were then incubated with secondary anti-mouse or anti-rabbit antibodies at room temperature for 1.5 h. The resulting immunoblots were visualized using LiCor/Odyssey infrared image system (LI-COR Biosciences, Lincoln, NE, USA). The intensity of each Western blot band was quantified and analysed using ImageJ software (NIH, Bethesda, MD, USA).

### RNA Extraction and Real-time PCR

Total RNA was isolated from tissues or HK2 cells the Trizol reagent (Invitrogen) according to the manufacturer's instructions. Real-time polymerase chain reaction was performed using BioRadiQ SYBR Green supermix with Opticon2 in a CFX96 Real-time PCR detection system, according to the manufacturer's instructions. Data were normalised to β-actin levels. The primers used are listed in Table S1 and Table S2.

### Co-IP

HK2 cells were seeded onto a dish and allowed to adhere to the cell wall for growth. After treatment with lead acetate, the cells were collected and Anti-HKDC1 antibody and magnetic beads were added. After overnight incubation, immune precipitants were detected by immunoblotting. Anti-rabbit and mouse IgG (#3900 and #3420; Cell Signaling Technology) were used as negative controls.

### Luciferase reporter assays

HK2 cells were seeded in 6-well plates and transfected with the wild-type or mutated HKDC1 plasmid. All cells were harvested 48 h after transfection and the firefly luciferase and Renilla luciferase activities in each well were calculated by a dual-luciferase reporter assay (Hanheng Biotechnology Co., Ltd., Shanghai, China). Firefly and Renilla Luciferase activities were examined using a Dual-Luciferase Reporter Assay System, and firefly activity was used to normalise the Renilla activity.

### Detection of ATP content

HK2 cells treated as indicated were lysed. ATP levels were detected using an enhanced ATP assay kit (Nanjing Jiancheng, China) according to the manufacturer's protocol.

### Detection of Lead content

An appropriate amount of mouse kidney tissue sample was weighed, and freshly prepared mixed digestion solution was added to it; after the sample was completely digested, it was placed on a temperature-controlled electric heating plate (200 ºC) to heat and drive acid until it reached a wet salt state. After slightly cooling, 1 mL of water was added to the sample, it was evaporated to a wet salt state, and digested completely; subsequently, the acid was removed. After cooling, the solution was diluted with 1 mL of 1% HNO3. The following settings were used for analysis: line wavelength of 283.3 nm; Lamp current 5mA; 0.4 nm slit; Carrier gas (Ar): 250 mL/min; Background correction: Deuterium lamp or Zeeman effect or self-absorption effect.

### Mass spectrometry analysis

Mass spectrometry (MS) analysis was conducted by OEbiotech (Shanghai, China). Briefly, the complexes bound to the immobilised magnetic beads were cleaned and digested using sequencing-grade modified trypsin. After extraction and purification, peptide samples were identified by mass spectrometry using a Q Exactive instrument (Thermo Finnigan, United). MaxQuant software (V1.6.2.10) was used to analyse raw mass spectrometry data.

### Statistical Analyses

All statistical analyses were performed using the SPSS version. The GraphPad Prism software package was used to generate figures. Differences among groups were tested using one-way or two-way ANOVA, followed by Tukey's post hoc test. Differences between the two groups were tested using an independent sample t-test. Results were expressed as mean ± SEM and as the number for categorical variables. All tests were two-sided, and *p* < 0.05 was considered statistically significant.

## Results

### M6A modification and methyltransferase METTL3 are highly induced in lead nephropathy *in vivo* and *in vitro*

To explore the pathogenesis and determine the treatment for lead nephropathy, an animal model of lead nephropathy was established according to the schedule shown in Figure S1A. C57/BL6J mice were intraperitoneally injected with lead acetate for 1, 2, or 3 weeks, and the best experimental conditions were determined by measuring renal function indicators, pathological changes, and kidney injury molecule. The results showed that the renal function of mice injected with lead acetate intraperitoneally for two weeks was seriously damaged, and the serum creatinine and urea nitrogen levels were significantly increased (Figure S1B and C). The results of PAS staining suggested that the renal tubules in mice were dilated and disordered after two weeks of exposure. (Figure S1D). The results of Real-time PCR and Western blot showed that Pb significantly increased the expression of KIM-1, a renal injury molecule (Figure S1E and F). Therefore, two weeks was chosen as the modelling time for subsequent animal experiments. We then constructed a cell injury model using lead acetate. Previous studies have reported that Pb accumulates in renal tubular epithelial cells; therefore, we chose human renal tubular epithelial cells (HK2) for our research. First, MTT analysis showed that lead acetate had a significant effect on cell viability at 2.5 mM (Figure S1G). We explored the cell damage caused by lead acetate in detail and found that the best time for cell injury mediator KIM-1 protein and mRNA expression laid a solid foundation for the next experiment (Figure S1 H and 1I).

**Figure 1:**
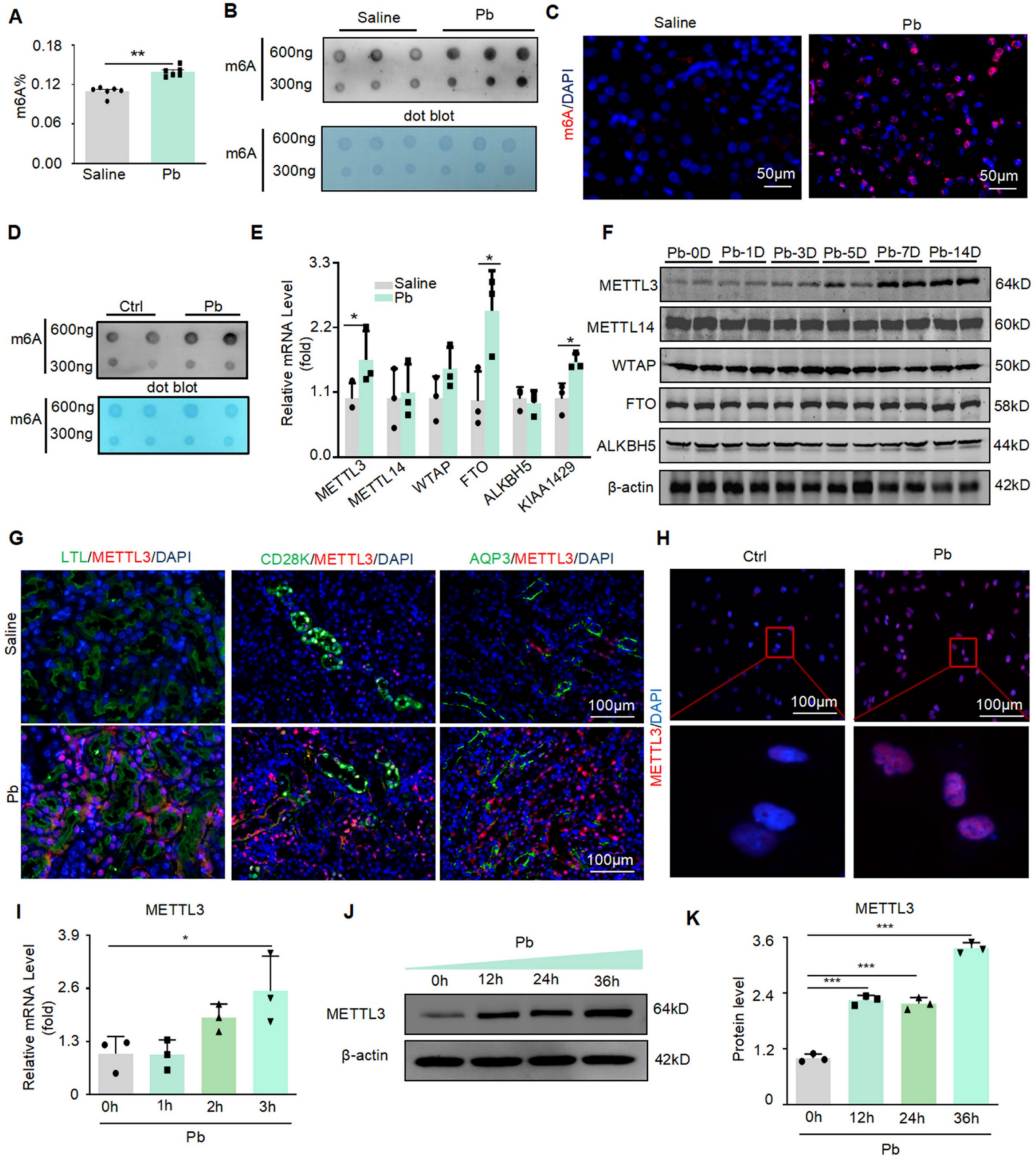
METTL3 mediated m6A modifications were significantly upregulated in lead nephropathy. (A) M6A enzyme-linked immunosorbent assay was used to assess m6A RNA methylation in mouse models of lead nephropathy (n = 6 biological replicates of mice, two tailed unpaired Student's t test). (B) Dot blot assay of m6A methylation modification abundance in lead nephropathy* in vivo*. (C) Representative immunofluorescence staining for m6A in the mouse model of lead nephropathy. Scale bars = 50 μm (D) Dot blot assay of m6A RNA methylation in HK2 cells induced by lead acetate* in vitro.* (E) Real-time PCR analysis of m6A regulators (METTL3, METTL14, WTAP, FTO, and ALKBH5) in a lead acetate-induced mouse model. (F) Western blot analysis of m6A regulators (METTL3, METTL14, WTAP, and FTO) induced by lead acetate in mice at different time points. (G) Representative immunofluorescence staining for METTL3, proximal tubular marker (LTL), distal tubule marker (CD28K), and collection pipe marker (AQP3) in a mouse model of lead nephropathy. Scale bars = 100 μm. (H) Representative immunofluorescence staining of METTL3 in Pb-treated HK2 cells. Scale bars = 100 μm. (I) Real-time PCR analysis of METTL3 mRNA expression in response to acetate stimulation (n = 3 biological replicates, one-way ANOVA with Tukey's multiple comparisons test). (J and K) Western blot analysis and quantitative assessment of METTL3 abundance induced by Pb (n = 3 biological replicates, one-way ANOVA with Tukey's multiple comparisons test). Data represent the mean ± S.E.M. *P< 0.05, **P< 0.01, ***P< 0.001.

In Pb-induced renal injury, we found that the total m6A level in mouse kidney tissue increased significantly after Pb treatment, using a rapid methylation detection kit and dot blot experiments (Figure 1A and B). Immunofluorescence further confirmed a significant increase in m6A levels after Pb treatment, mainly in the nucleus (Figure 1C). Similar results were obtained for the Pb-treated HK2 cells. Dot blotting showed that the m6A levels significantly increased after lead acetate treatment (Figure 1D). We then detected the mRNA expression of m6A methyltransferases (METTL3, METTL14, and WTAP) and demethyltransferases (FTO and ALKBH5) and found that METTL3 and FTO mRNA expression was upregulated in Pb-induced nephropathy compared with controls (Figure 1E). To further explore the specific time when lead acetate caused an increase in METTL3 in the kidney tissue, we injected lead acetate intraperitoneally for 1, 3, 5, 7, and 14 days and found that the protein abundance of METTL3 began to increase on the seventh day, and the protein abundance increased with time (Figure 1F). However, we did not detect consistent changes in FTO, METTL14, or WTAP protein abundance in lead nephropathy mouse models. Furthermore, immunofluorescence staining of METTL3 in different segments of renal tubules from kidney tissues of lead nephropathy mice showed that METTL3 was not only hyperexpressed in the proximal tubule region (LTL) but was also significantly elevated in the collecting duct (AQP3) and distal duct (CD28K) compared to the kidney tissues of the control group (Figure 1G). And located within the nucleus of the cell (Figure 1H). We also found that METTL3 mRNA expression was induced in HK2 cells in response to Pb (Figure 1I). Furthermore, METTL3 protein abundance increased in a time-dependent manner in the Pb-induced cell injury model (Figure 1 J and K).

### METTL3 deletion protects against lead acetate-induced renal injury and inflammation in mice

**Figure 2:**
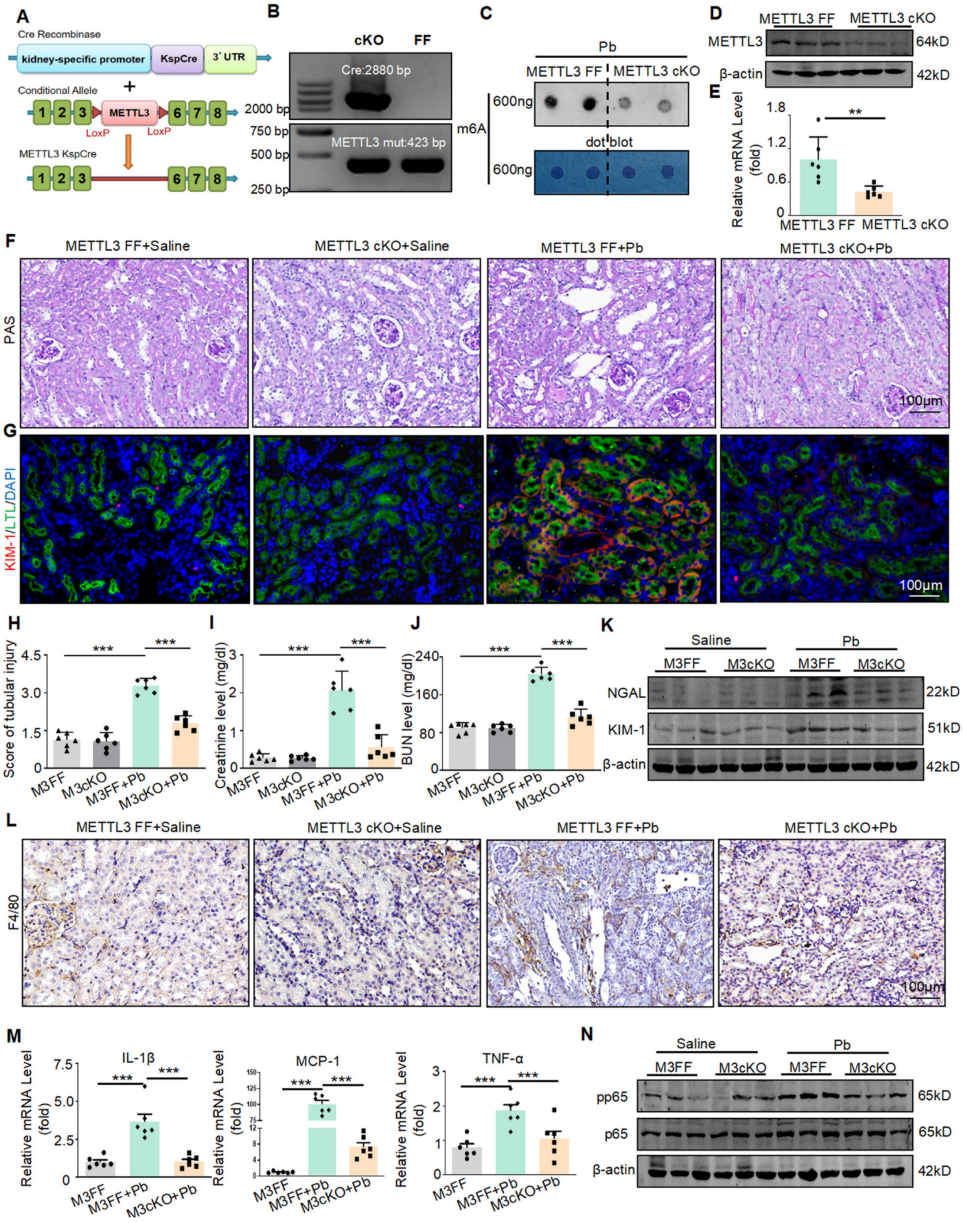
METTL3 cKO protects against renal dysfunction and inflammation induced by lead acetate. (A) Schematic representation of the genetic approach used to generate METTL3 conditional knockout (cKO) mice. (B) METTL3 deficiency was confirmed using genomic DNA analysis. (C) Dot blot assay of m6A methylation modification abundance in lead nephropathy with or without METTL3 cKO. (D) Western blot analysis of METTL3 abundance in METTL3 cKO mice compared to METTL3^Flox/Flox^ mice. (E) Real-time PCR analysis of METTL3 mRNA expression in METTL3 cKO mice compared to METTL3^Flox/Flox^ mice (n = 6 biological replicates of mice, two tailed unpaired Student's t test). (F and H) Representative PAS staining picture and quantification analysis of kidneys from METTL3^Flox/Flox^ and METTL3 cKO mice treated with lead acetate. Scale bars = 100 μm. (n = 6 biological replicates, one-way ANOVA with Tukey's multiple comparisons test). (G) Representative immunofluorescence staining of KIM-1 and proximal tubular markers (LTL). Scale bars = 100 μm. (I and J) Serum creatinine and BUN levels in METTL3^Flox/Flox^ and METTL3 cKO mice exposed to Pb (n = 6 biological replicates, one-way ANOVA with Tukey's multiple comparisons test). (K) Western blot analysis of KIM-1 and NGAL levels in METTL3^Flox/Flox^ and METTL3 cKO mice exposed to Pb. (L) IHC staining for F4/80+ macrophage infiltration in METTL3^Flox/Flox^ and METTL3 cKO mice with lead nephropathy. (M) Real-time PCR analysis of inflammatory cytokines (TNF-α, MCP-1 and IL-1β) in lead acetate-induced lead nephropathy (n = 6 biological replicates, one-way ANOVA with Tukey's multiple comparisons test). (N) Western blot analysis of p65 NF-κB phosphorylation in lead nephropathy. Data represents the mean ± S.E.M. **P< 0.01, ***P< 0.001. M3FF : METTL3^Flox/Flox^ , M3cKO : METTL3 cKO.

To confirm the functions of METTL3 *in vivo*, we successfully constructed Ksp-Cre METTL3^fl/fl^ mice using the Cre-LoxP recombination system and generated METTL3 cKO mice (Figure 2A). All the mice were genotyped using PCR (Figure 2B), and it was found that the conditional knockout of METTL3 reduces m6A methylation levels (Figure 2C). The absence of METTL3 in TECs was verified by Real-time PCR and Western blot (Figure 2D and 2E). Two weeks after lead acetate injection, serum creatinine and urea nitrogen levels, which are serological indicators of renal function, were significantly increased, whereas METTL3 cKO significantly reduced the abnormal elevation of these indicators (Figure 2I and J). PAS staining and quantitative analysis showed that METTL3 cKO attenuated Pb-induced renal tubular dilatation and other pathological changes (Figure 2F and 2H). The protein levels of the renal tubular injury markers (KIM-1 and NGAL) were increased in Pb-induced nephropathy and downregulated in METTL3 cKO mice (Figure 2G and Figure 2K and Figure S2A and S2B).

In addition to renal injury, an inflammatory response also occurs. Therefore, we detected the expression of inflammatory factors and found that lead acetate can increase the expression of proinflammatory cytokine production (TNF-α, IL-1β and MCP-1) After knocking out METTL3, this elevation was alleviated (Figure 2M). In addition, immunohistochemistry and Western blot showed that the absence of METTL3 reduced the infiltration of F4/80+macrophages (Figure 2L) and inhibited activation of p65 NF-κB (Figure 2N and Figure S2C). However, after testing the lead content in the kidney tissues of both METTL3 Flox/Flox and METTL3 cKO mice treated with lead acetate, we found that METTL3 cKO did not reduce the accumulation of lead in the kidneys (Figure S2D). In summary, these results indicated that METTL3 is a key mediator of renal injury and inflammation induced by lead acetate.

### Silencing METTL3 reduces the cell injury and inflammatory response induced by lead acetate *in vitro*

**Figure 3:**
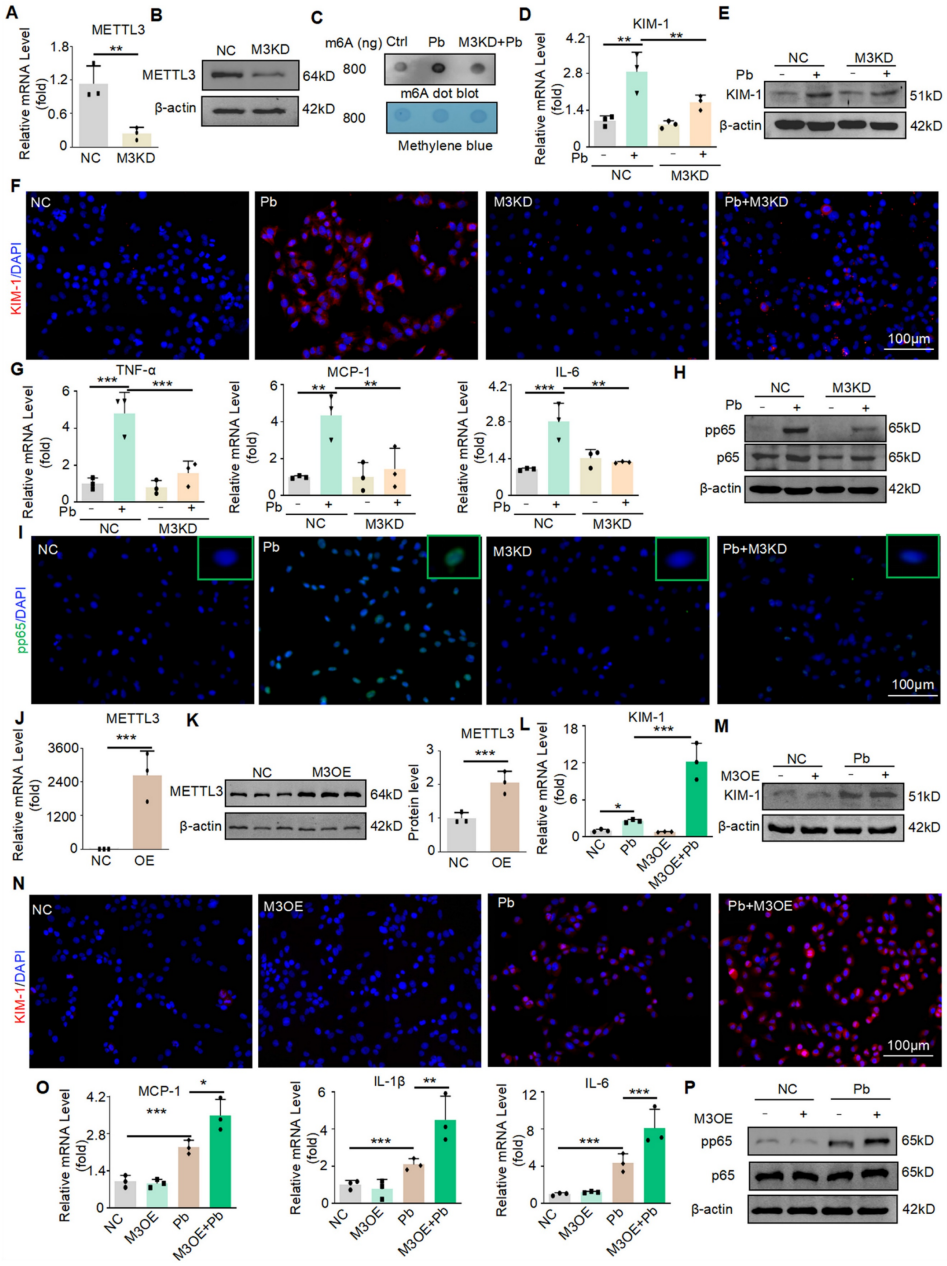
METTL3 enhances cell damage and inflammatory response in HK2 cells treated with lead acetate. (A) METTL3 knockdown was confirmed by Real-time PCR analysis (n = 3 biological replicates, two tailed unpaired Student's t test). (B) METTL3 knockdown was confirmed by Western blot analysis. (C) Dot blot assay of m6A methylation in lead acetate-treated METTL3 knockdown HK2 cells. (D) Real-time PCR analysis of KIM-1 expression in lead acetate-treated HK2 cells with and without METTL3 knockdown. (n = 3 biological replicates, one-way ANOVA with Tukey's multiple comparisons test). (E) Western blot analysis of KIM-1 in lead acetate-treated HK2 cells with and without METTL3 knockdown. (F) Representative immunofluorescence staining of KIM-1 in lead acetate-treated HK2 cells with and without METTL3 knockdown. Scale bars = 100 μm. (G) Real-time PCR analysis of expression of inflammatory cytokines and chemokine (TNF-α, IL-6, and MCP-1) in lead acetate-treated HK2 cells with and without METTL3 knockdown (n = 3 biological replicates, one-way ANOVA with Tukey's multiple comparisons test). (H) Western blot analysis of pp65 and p65 in lead acetate-treated HK2 cells with or without METTL3 knockdown. (I) Representative immunofluorescence staining of pp65 in lead acetate-treated HK2 cells with and without METTL3 knockdown. Scale bars = 100 μm. (J) METTL3 overexpression was confirmed using Real-time PCR (n = 3 biological replicates, two tailed unpaired Student's t test). (K) METTL3 Overexpression was confirmed by Western blot. (L) Real-time PCR analysis of KIM-1 expression in lead acetate-treated HK2 cells with and without METTL3 overexpression (n = 3 biological replicates, one-way ANOVA with Tukey's multiple comparisons test). (M) Western blot analysis of KIM-1 in lead acetate-treated HK2 cells with and without METTL3 overexpression. (N) Representative immunofluorescence staining of KIM-1 in lead acetate-treated HK2 cells with and without METTL3 overexpression. Scale bars = 100 μm. (O) Real-time PCR analysis of expression of inflammatory cytokines and chemokine (IL-1β, IL-6, and MCP-1) in lead acetate-treated HK2 cells with and without METTL3 overexpression (n = 3 biological replicates, one-way ANOVA with Tukey's multiple comparisons test). (P) Western blot analysis of pp65 and p65 in METTL3 knockdown HK2 cells with or without METTL3 overexpression. Data represents the mean ± S.E.M. *P< 0.05, **P< 0.01, ***P< 0.001. M3KD : METTL3 knockdown. M3OE : METTL3 overexpression.

Next, we evaluated the detailed function of METTL3 in lead acetate-treated HK2 cells. Knockdown of METTL3 by siRNA transfection in HK2 cells was verified by Real-time PCR and Western blot (Figure 3A and 3B). The m6A modification was markedly enhanced in the lead acetate-induced cell injury model compared to the control, whereas knockdown of METTL3 attenuated the abnormally increased level of m6A modification. This showed that METTL3 plays an important role in the m6A methylation process in the lead acetate-induced cell injury model (Figure 3C). Moreover, the mRNA and protein levels of KIM-1 were reduced after METTL3 knockdown in the lead acetate-induced cell injury model (Figure 3D and 3E). This result was confirmed by immunofluorescence staining (Figure 3F). Real-time PCR and Western blot analyses demonstrated that knockdown of METTL3 suppressed mRNA levels of proinflammatory cytokines (TNF-α and IL-6) and chemokines (MCP-1), and protein levels of the p65 nuclear factor κB (p65 NF-κB) phosphorylation compared with lead acetate-stimulated HK2 cells (Figure 3G and 3H). METTL3 knockdown can also inhibit p65 phosphorylation into the nucleus induced by lead acetate as demonstrated by immunofluorescence staining (Figure 3I). Subsequently, we transfected the cells with METTL3 overexpression plasmids and successfully overexpressed them using Real-time PCR and Western blot (Figure 3J and 3K). Contrary to the silencing of METTL3, the overexpression of METTL3 increased the mRNA and protein levels of KIM-1 in the lead acetate-induced cell injury model (Figure 3L, 3M, and 3N). Real-time PCR and Western blot analyses demonstrated that overexpression of METTL3 promoted mRNA levels of proinflammatory cytokines (IL-6 and IL-1β) and chemokines (MCP-1), and protein levels of the p65 nuclear factor κB (p65 NF-κB) phosphorylation compared with lead acetate-stimulated HK2 cells (Figure 3O and 3P). Taken together, these results indicate that METTL3 is a crucial mediator of lead acetate-induced HK2 cell injury and inflammatory reactions.

### HKDC1 is a target of METTL3, and METTL3 enhances the HKDC1 mRNA stability in an IGF2BP2 dependent manner

**Figure 4:**
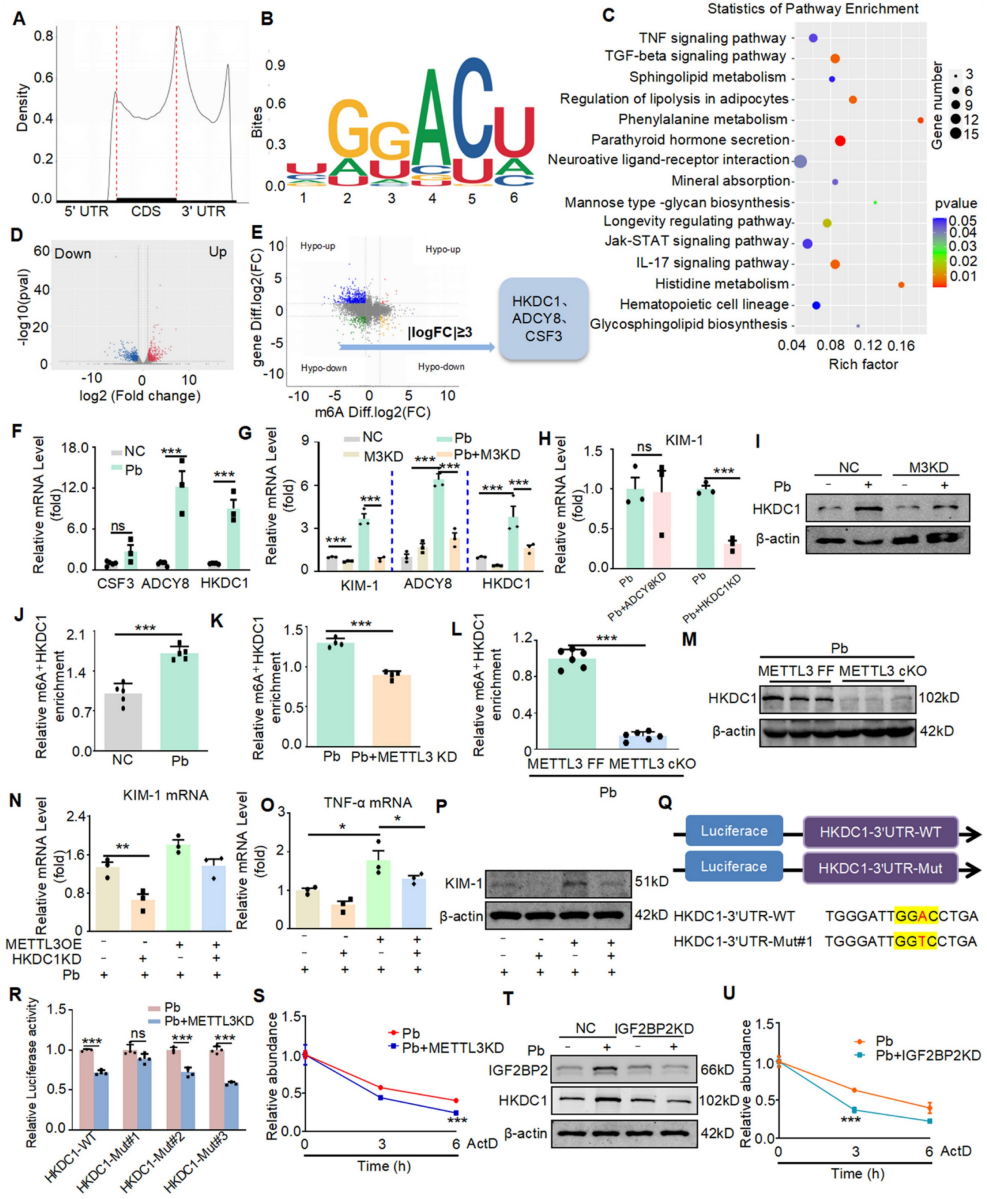
HKDC1 serves as a target of METTL3 via an IGF2BP2-dependent mechanism. (A) Density distribution of m6A peaks across the mRNA transcripts. (B) Predominant consensus motif “GGAC” was detected in METTL3 cKO mice and METTL3^Flox/Flox^ exposed to lead acetate. (C) KEGG enrichment analysis of METTL3-mediated m6A modifications. (D) Volcano map of differential expression genes in METTL3 cKO mice and METTL3^Flox/Flox^ exposed to lead acetate. (E) Filtering of the diminished m6A peaks with differentially expressed genes in METTL3 cKO mice and METTL3^Flox/Flox^ exposed to lead acetate identified HKDC1 and ADCY8 as direct targets of METTL3. (F) Verification of potential targets by real-time PCR in lead acetate-stimulated HK2 cells (n = 3 biological replicates, two tailed unpaired Student's t test). (G) Verification of potential targets in METTL3 knockdown HK2 cells stimulated with Pb acetate (n = 3 biological replicates, one-way ANOVA with Tukey's multiple comparisons test). (H) Real-time PCR analysis of KIM-1 in lead acetate-stimulated HK2 cells after ADCY8 KD and HKDC1 KD (n = 3 biological replicates, two tailed unpaired Student's t test). (I) Western blot analysis of HKDC1 after METTL3 knockdown in Pb -treated HK2 cells. (J) MeRIP-qPCR analysis of alterations in m6A modifications of HKDC1 genes in lead acetate-stimulated HK2 cells (n = 5 biological replicates, two tailed unpaired Student's t test). (K) MeRIP-qPCR analysis of alterations in m6A modifications of HKDC1 genes in lead acetate-stimulated HK2 cells with or without METTL3 knockdown. (n = 4 biological replicates, two tailed unpaired Student's t test). (L) MeRIP-qPCR analysis of alterations in m6A modifications of HKDC1 genes in lead acetate-treated METTL3^Flox/Flox^ mice and METTL3 cKO mice (n = 6 biological replicates, two tailed unpaired Student's t test). (M) Western blot analysis of HKDC1 in lead acetate-treated METTL3^Flox/Flox^ mice and METTL3 cKO mice. (N) Real-time PCR analysis of KIM-1 in METTL3-overexpression HK2 cells with HKDC1 knockdown (n = 3 biological replicates, one-way ANOVA with Tukey's multiple comparisons test). (O) Real-time PCR analysis of TNF-α in METTL3-overexpression HK2 cells with HKDCI knockdown (n = 3 biological replicates, one-way ANOVA with Tukey's multiple comparisons test). (P) Western blot analysis of KIM-1 in METTL3-overexpression HK2 cells with HKDC1 knockdown. (Q) Schematic of the methylation modification site in HKDC1. (R) Luciferase reporter assay measured the luciferase activities of HKDC1 WT or HKDC1 Mut in Pb-treated HK2 cells with or without METTL3 knockdown. (S) The decay rate of HKDC1 mRNA after administration of actinomycin D (5 μg/mL) in METTL3 knockdown HK2 cells. (T) Western blot analysis of HKDC1 after IGF2BP2 inhibition in lead acetate-treated HK2 cells. (U) The decay rate of HKDC1 mRNA after actinomycin D (5 μg/mL) administration in IGF2BP2 knockdown HK2 cells. Data represents the mean ± S.E.M. *P< 0.05, **P< 0.01, ***P< 0.001. M3KD : METTL3 knockdown.

To further explore the specific molecular mechanism of promoting injury and inflammation effects of METTL3, we performed the methylated RNA immunoprecipitation sequencing (MeRIP-seq) and RNA-Seq analysis of cKO METTL3 mouse kidney tissue samples treated with lead acetate and wild-type mouse kidney tissue samples treated with lead acetate. According to the MeRIP-seq analysis, m6A peaks were particularly abundant in the vicinity of 3′ untranslated regions (3′UTRs) near stop codons (Figure 4A), and m6A modifications were typically located in a consensus “GGAC” motif (Figure 4B). The analysis of Kyoto Encyclopedia of Genes and Genomes (KEGG) enrichment indicated that the methylation of m6A was closely related to the inflammatory pathway (such as the TNF-α pathways), which was further supporting the role of METTL3 in inflammation, and consistent with previous studies (Figure 4C). What are the potential downstream targets for METTL3 regulation. MeRIP-seq analysis revealed that 915 peaks decreased and 677 peaks increased in Pb-induced nephropathy in METTL3 cKO mice compared with Flox/Flox mice. In the RNA-seq analysis, 799 genes were downregulated and 768 genes were upregulated (Figure 4D). Compared with Pb-induced nephropathy in METTL3 Flox/Flox mice, correlation analysis of mRNA and m6A modifications showed that 3909 genes were significantly upregulated, whereas 2978 genes were significantly downregulated in cKO METTL3 mice (GSE288167). Importantly, we performed intersection analysis of the mRNA and m6A sequencing results with a fold change (FC) ≥ 3 and a p-value < 0.05 as the filtering criteria using Venn diagrams.

After excluding logical faults, we screened three potential genes (Figure 4E). Real-time PCR revealed increased expression of two genes, ADCY8 and HKDC1, which is consistent with the RNA-seq (Figure 4F). Validation of METTL3 cKO kidney tissues using Real-time PCR revealed two genes that showed consistent expression changes in each group, based on the sequencing results (Figure 4G). We selected ADCY8 and HKDC1 genes to explore their potential functions. Initially, we screened appropriate siRNAs for these potential genes using Real-time PCR (Figure S3A and S3B). We then silenced two potential genes in Pb-treated HK2 cells and found that knockdown of HKDC1, but not the other genes, attenuated Pb-induced cell injury (Figure 4H). Moreover, the MeRIP-seq results showed that the m6A methylation level of HKDC1 decreased most significantly after METTL3 cKO (Figure S3C). Western blot further confirmed that HKDC1 upregulation was inhibited after METTL3 knockdown in HK2 cells treated with lead acetate (Figure 4I). Subsequently, using MeRIP-qPCR, we found that the m6A antibody indeed enriched HKDC1 mRNA in HK2 cells treated with lead acetate (Figure 4J). Moreover, the m6A methylation of HKDC1 mRNA induced in HK2 cells treated with lead acetate was inhibited by METTL3 silencing (Figure 4K). In addition, we found that METTL3 cKO reduced the enrichment of m6A antibodies in HKDC1 mRNA and decreased the protein expression of HKDC1 (Figure 4L and 4M). Western blot and Real-time PCR experiments revealed that rescuing METTL3 expression in HKDC1 knockdown HK2 cells did not exacerbate lead acetate-induced cell injury and inflammatory reactions (Figure 4N, 4O, and 4P). To further verify that METTL3 targets HKDC1 mRNA for m6A modification, we constructed luciferase reporters containing either wild-type or mutant HKDC1 (Figure 4Q and S3D). In mutant HKDC1, m6A modification was abrogated by replacing adenine with thymine in the m6A consensus sequences (RRACH). A luciferase reporter assay showed that the amount of the wild-type HKDC1 transcript, but not the mutant gene, decreased in the absence of METTL3 (Figure 4R). These results suggested that the regulation of HKDC1 was under the control by METTL3-associated m6A modifications. Hence, these results indicated that HKDC1 is a direct target gene of METTL3-mediated m6A modification in lead nephropathy.

We further explored the mechanism of post-transcriptional regulation of HKDC1 mRNA via the interaction between METTL3 and m6A binding protein. We used a transcriptional inhibitor (actinomycin D) and Real-time PCR to determine the degradation rate of HKDC1 mRNA. The results showed that in Pb-stimulated HK2 cells, METTL3 deficiency significantly reduced the degradation half-life of HKDC1 mRNA, indicating that METTL3 mediated m6A modification promotes HKDC1 mRNA stability (Figure 4S). Considering the role of reading proteins in the process of m6A modification and the non-negligible role of IGF2BP family members in regulating the translation and stability of m6A-modified mRNAs, we examined the involvement of IGF2BP1, IGF2BP2, and IGF2BP3 in HKDC1 mRNA stability. For IGF2BP1, IGF2BP2, and IGF2BP3, we designed two siRNAs and confirmed their interference efficiency using Real-time PCR (Figure S3E). Moreover, the silencing of IGF2BP2, but not IGF2BP1 or IGF2BP3, significantly inhibited the mRNA and protein expression of HKDC1 (Figure S3F and S3G). Next, RNA stability assays also showed that silencing IGF2BP2 followed by treatment with actinomycin D (5 μg/mL) significantly reduced the degradation half-life of HKDC1 mRNA in lead-treated HK2 cells (Figure 4U), suggesting that IGF2BP2 plays a critical role in the maintenance of HKDC1 mRNA stability. Western blot analysis indicated that IGF2BP2 knockdown inhibited HKDC1 protein expression (Figure 4T). And IGF2BP2 knockdown could also alleviate the damage and inflammatory response of renal tubular epithelial cells caused by lead acetate (Figure S3H and S3I). Collectively, these results suggested that METTL3-dominated HKDC1 m6A modification enhanced mRNA stability and protein expression, with IGF2BP2 playing an indispensable auxiliary role.

### Disruption of HKDC1 alleviates lead acetate-induced renal injury and inflammatory response *in vitro* and *in vivo*

**Figure 5:**
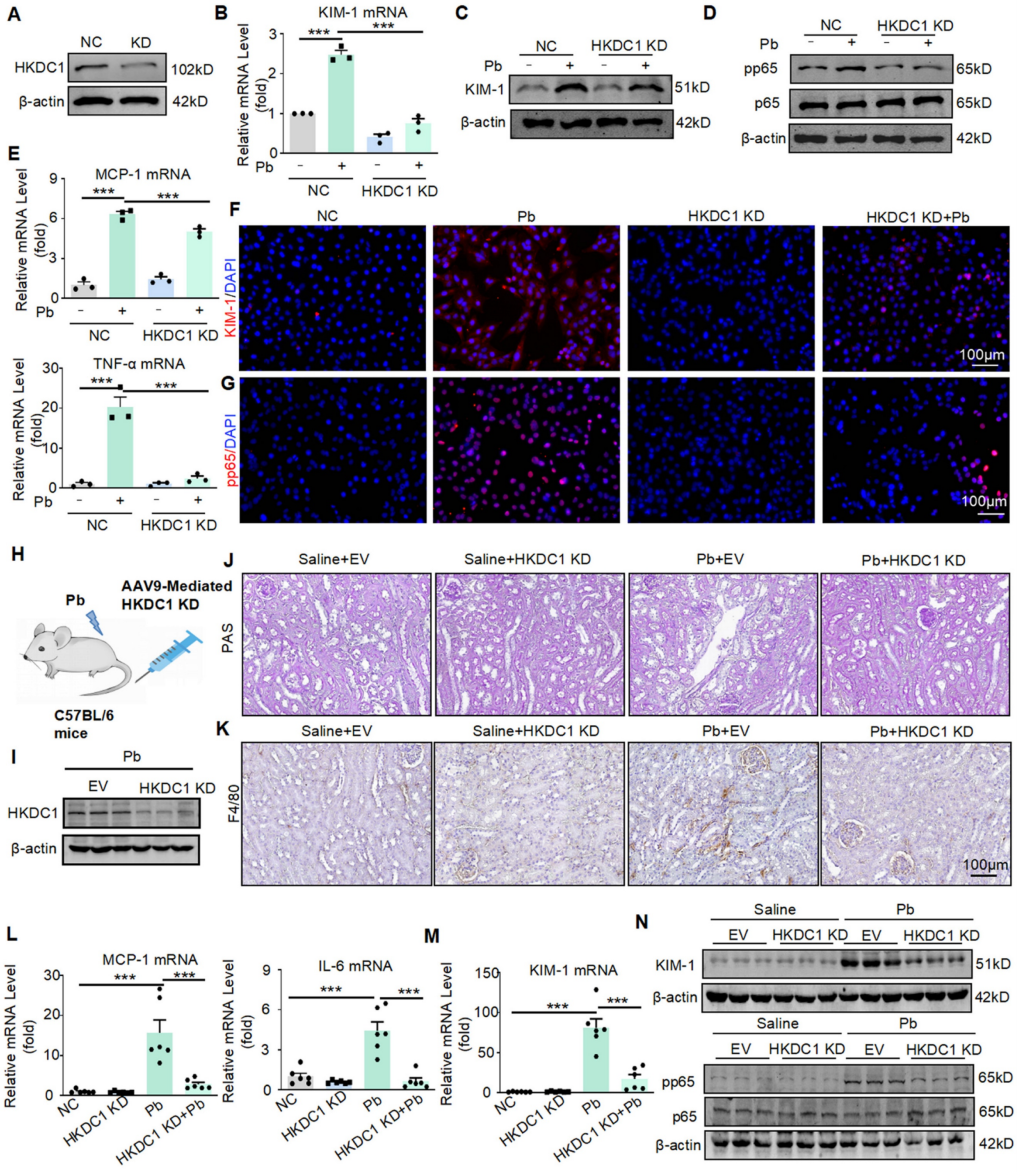
Silencing HKDC1 attenuates renal injury and inflammatory response in* vitro* and* in vivo*. (A) HKDC1 knockdown was confirmed by Western blotting. (B) Real-time PCR analysis of KIM-1 expression in lead acetate-treated HK2 cells with and without HKDC1 knockdown (n = 3 biological replicates, one-way ANOVA with Tukey's multiple comparisons test). (C) Western blot analysis of KIM-1 in lead acetate-treated HK2 cells with and without HKDC1 knockdown. (D) Western blot analysis of pp65 and p65 in lead acetate-treated HK2 cells with or without HKDC1 knockdown. (E) Real-time PCR analysis of expression of inflammatory cytokines and chemokine (MCP-1 and TNF-α) in lead acetate-treated HK2 cells with and without HKDC1 knockdown (n = 3 biological replicates, one-way ANOVA with Tukey's multiple comparisons test). (F) Representative immunofluorescence staining of KIM-1 in lead acetate-treated HK2 cells with and without HKDC1 knockdown. Scale bars = 100 μm. (G) Representative immunofluorescence staining of pp65 in lead acetate-treated HK2 cells with and without HKDC1 knockdown. Scale bars = 100 μm. (H) Schematic diagram of AAV9 mediated HKDC1 silencing by tail vein injection. (I) The expression of HKDC1 in mice. (J) PAS staining to detect tubular injury in Pb-treated mice with or without HKDC1 knockdown. Scale bars = 100 μm. (K) IHC staining of F4/80+ macrophage infiltration in lead acetate-treated mouse kidneys. Scale bars = 100 μm. (L) Real-time PCR analysis of MCP-1 and IL-6 mRNA expression in lead acetate-treated HKDC1 knockdown mice (n = 6 biological replicates, one-way ANOVA with Tukey's multiple comparisons test). (M) Real-time PCR analysis of KIM-1 mRNA expression in lead acetate-treated mice with HKDC1 knockdown (n = 6 biological replicates, one-way ANOVA with Tukey's multiple comparisons test). (N) Western blot analysis of p65 phosphorylation and KIM-1 expression in lead acetate-treated mice with HKDC1 knockdown. Data represents the mean ± S.E.M. ***P< 0.001. HKDC1 KD: HKDC1 knockdown.

To investigate the functional role of HKDC1, we silenced HKDC1 using siRNA in HK2 cells and validated the silencing efficiency using Western blot (Figure 5A). Real-time PCR and Western blot showed that HKDC1 knockdown reduced the mRNA and protein expression of KIM-1 induced by lead acetate in HK2 cells (Figure 5B and 5C). Immunofluorescence further confirmed that HKDC1 knockdown reduced the abnormal expression of KIM-1 induced by lead acetate in HK2 cells (Figure 5F). Moreover, HKDC1 knockdown decreased the lead acetate-induced activation of the p65 NF-κB activation (Figure 5D and 5G). Real-time PCR showed that HKDC1 knockdown reduced the expression of TNF-α and MCP-1 induced by lead acetate in HK2 cells (Figure 5E). To determine the function of HKDC1* in vivo*, we silenced HKDC1 in mice using an AAV9-packaged HKDC1 knockdown plasmid (Figure 5H).

Western blot confirmed that AAV9-packaged HKDC1 knockdown plasmid reduced the expression of HKDC1 (Figure 5I). Knockdown of HKDC1 prevented lead acetate-induced renal damage confirmed by PAS staining (Figure 5J). Furthermore, the expression of KIM-1 was decreased at both the protein and mRNA levels after HKDC1 knockdown (Figure 5M and 5N). Mechanistically, knockdown of HKDC1 inhibited p65 NF-κB activation confirmed by Western blot (Figure 5N). Real-time PCR and immunohistochemistry analysis showed that the knockdown of HKDC1 reduced the expression of IL-6, MCP-1 and F4/80+ macrophage infiltration (Figure 5L and 5K).

### HKDC1 binds to the ATPB and inhibits ATPB ubiquitination by antagonizing E3 ligase Murf1 to promote injury and inflammatory response

**Figure 6:**
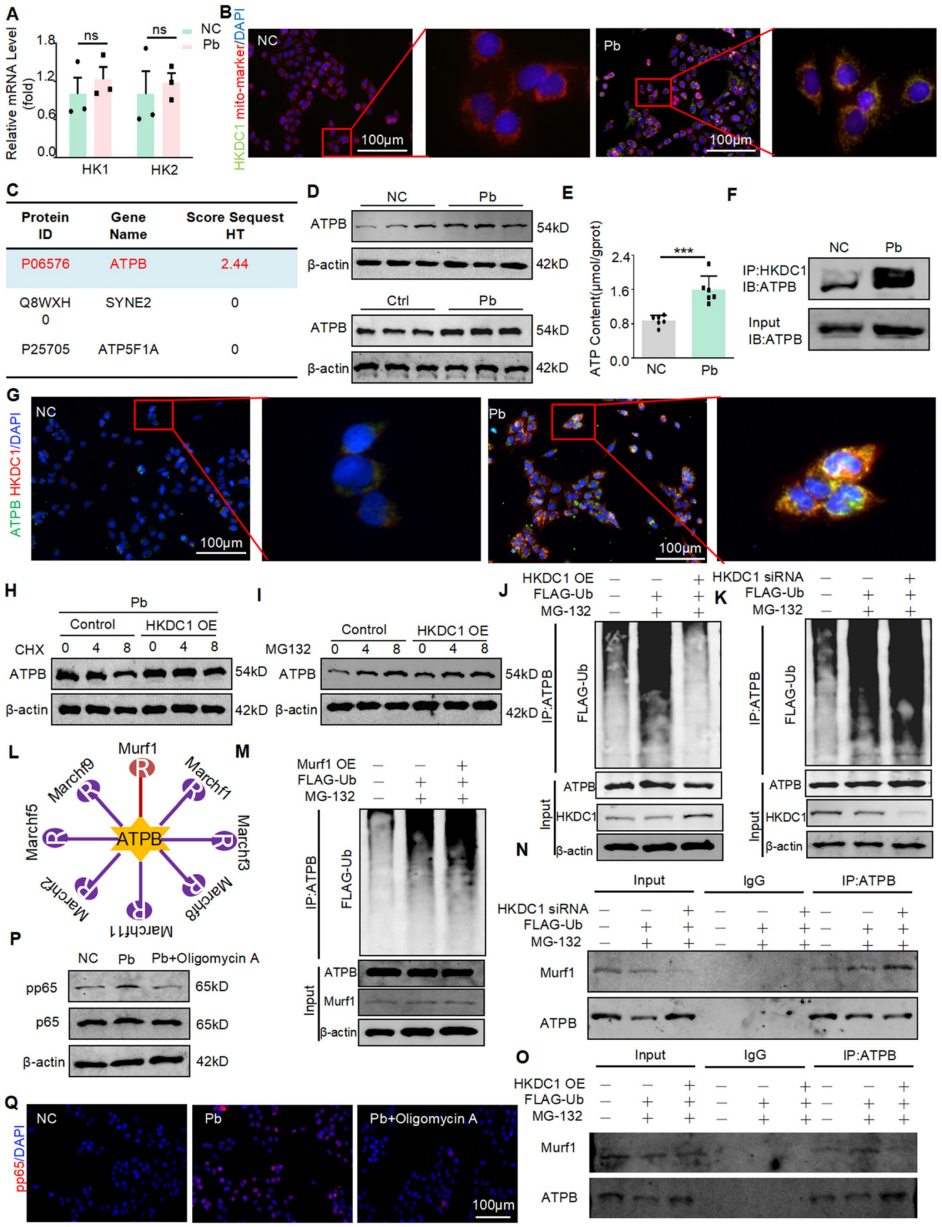
HKDC1 inhibits ATPB binding by antagonizing E3 ligase Murf1, leading to ubiquitination and promoting ATP production. (A) Real-time PCR analysis of HK1 and HK2 in Pb-treated HK2 cells (n = 3 biological replicates, two tailed unpaired Student's t test). (B) Representative immunofluorescence staining of HKDC1 and mito-marker in Pb-treated HK2 cells. Scale bars = 100 μm. (C) Liquid chromatography-mass spectrometry. (D) Western blot analysis of ATPB in lead acetate-treated HK2 cell and mice. (E) ATP content of lead acetate-treated HK2 cells (n = 6 biological replicates, two tailed unpaired Student's t test). (F) Co-immunoprecipitation (co-IP) assay to detect interactions between HKDC1 and ATPB in lead acetate-treated HK2 cells. (G) Representative immunofluorescence staining of HKDC1 and ATPB in Pb-treated HK2 cells. Scale bars = 100 μm. (H) ATPB expression was assessed after different durations of CHX administration in HKDC1-OE HK2 cells. (I) ATPB expression was assessed after different durations of MG132 administration in HKDC1 OE HK2 cells. (J) HKDC1 OE and FLAG-Ub were co-expressed and applied with or without MG132 in HK2 cells. (K) HKDC1 knockdown and FLAG-Ub co-expression and treatment with or without MG132 in HK2 cells. (L) The UbiBrowser assay. (M) Murf1 overexpression and FLAG-Ub co-expression and treatment with or without MG132 in HK2 cells. (N) HKDC1 knockdown and FLAG-Ub co-expression and treatment with or without MG132 in HK2 cells. (O) HKDC1 OE and FLAG-Ub co-expression and treatment with or without MG132 in HK2 cells. (P) Western blot analysis of p65 NF-κB phosphorylation in ATPB inhibitor Oligomycin A-treated mice in response to lead acetate. (Q) Representative immunofluorescence staining of pp65 in lead acetate-treated HK2 cells with and without Oligomycin A. Scale bars = 100 μm. Data represents the mean ± S.E.M. ***P< 0.001. HKDC1 OE: HKDC1 overexpression.

HKDC1 is a member of the HK family, which includes HK1-2 and HKDC1. To determine whether HKDC1 is specifically regulated by METTL3, we examined the expression of HK1-2 mRNA in HK2 cells with or without lead acetate treatment. However, the expression levels of HK1 and HK2 were not significantly different after treatment with lead acetate (Figure 6A). Subsequently, the co-localisation of HKDC1 with mitochondrial markers was discovered by immunofluorescence staining (Figure 6B). To investigate whether HKDC1 synergistically regulates damage and inflammatory responses with specific proteins, we conducted co-IP assays and performed mass spectrometry. According to the MS Prot score and localisation, ATPB was the most likely interacting protein of HKDC1 (Figure 6C). As an ATP synthase subunit, ATPB is expressed in the mitochondrial membrane and is responsible for ATP production. We first determined the ATPB expression in lead nephropathy *in vitro* and *in vivo* using Western blot. Interestingly, ATPB expression was upregulated after treatment with lead acetate (Figure 6D). In addition, ATP concentration was altered (Figure 6E). The interaction between HKDC1 and ATPB was further confirmed by immunofluorescence and immunoblotting (Figure 6F and 6G). To determine whether HKDC1 increases the expression of ATPB by promoting ATPB transcription or inhibiting ATPB proteasomal degradation, the protein synthesis inhibitor CHX and the proteasome inhibitor MG132 were used to determine the effects of HKDC1 on ATPB in HK2 cells. Compared with the HKDC1 OE group, the control group displayed markedly reduced expression of ATPB in a CHX time-dependent manner, suggesting that HKDC1 does not affect ATPB protein synthesis. Moreover, the expression of ATPB increased gradually with increasing duration of MG132 stimulation in both the control and HKDC1 OE groups. However, with MG132 stimulation, the increased induction and level of ATPB expression in the control group were greater than those in the HKDC1 OE group (Figure 6H and 6I). These results suggest that HKDC1 increases ATPB expression by inhibiting proteasome-mediated ATPB degradation. To determine whether HKDC1 induces ATPB production via ATPB polyubiquitination, Co-IP was performed to determine the effect of HKDC1 on ATPB ubiquitination in HK2 cells. The results show that HKDC1 OE significantly decreased ATPB polyubiquitination in the presence of MG132. In contrast, HKDC1 knockdown increased ATPB polyubiquitination in the presence of MG132 (Figure 6J and 6K). To further explore the influence of HKDC1 on ATPB ubiquitination, UbiBrowser (http://ubibrowser.ncpsb.org) was used to predict the ubiquitinated region of ATPB (Figure 6L). The results revealed that the large overlap of the ubiquitin E3 ligases Murf1 and HKDC1 with the ATPB-binding region SUMOylate ATPB-1 could be targeted by the poly SUMO-specific ubiquitin E3 ligase Murf1 and subsequently tagged for degradation by the ubiquitin proteasome system. Consistent with these results, Murf1 overexpression significantly increased ATPB polyubiquitination in the presence of MG132 (Figure 6M). Additionally, our results showed that HKDC1 downregulation increased the interaction between ATPB and Murf1 in the presence of MG132 (Figure 6N). In contrast, HKDC1 overexpression significantly reduced the intensity of the interaction between ATPB and Murf1 in the presence of MG132 (Figure 6O). Oligomycin A is an inhibitor of ATPB, Western blot and immunofluorescence results showed that Oligomycin A inhibited the phosphorylation of NF-кB (p65) (Figure 6P and 6Q). These findings indicate that HKDC1 inhibits ATPB ubiquitination by antagonising the E3 ligase Murf1.

### AAV9-mediated silencing of METTL3 protects against lead acetate-induced renal injury

**Figure 7:**
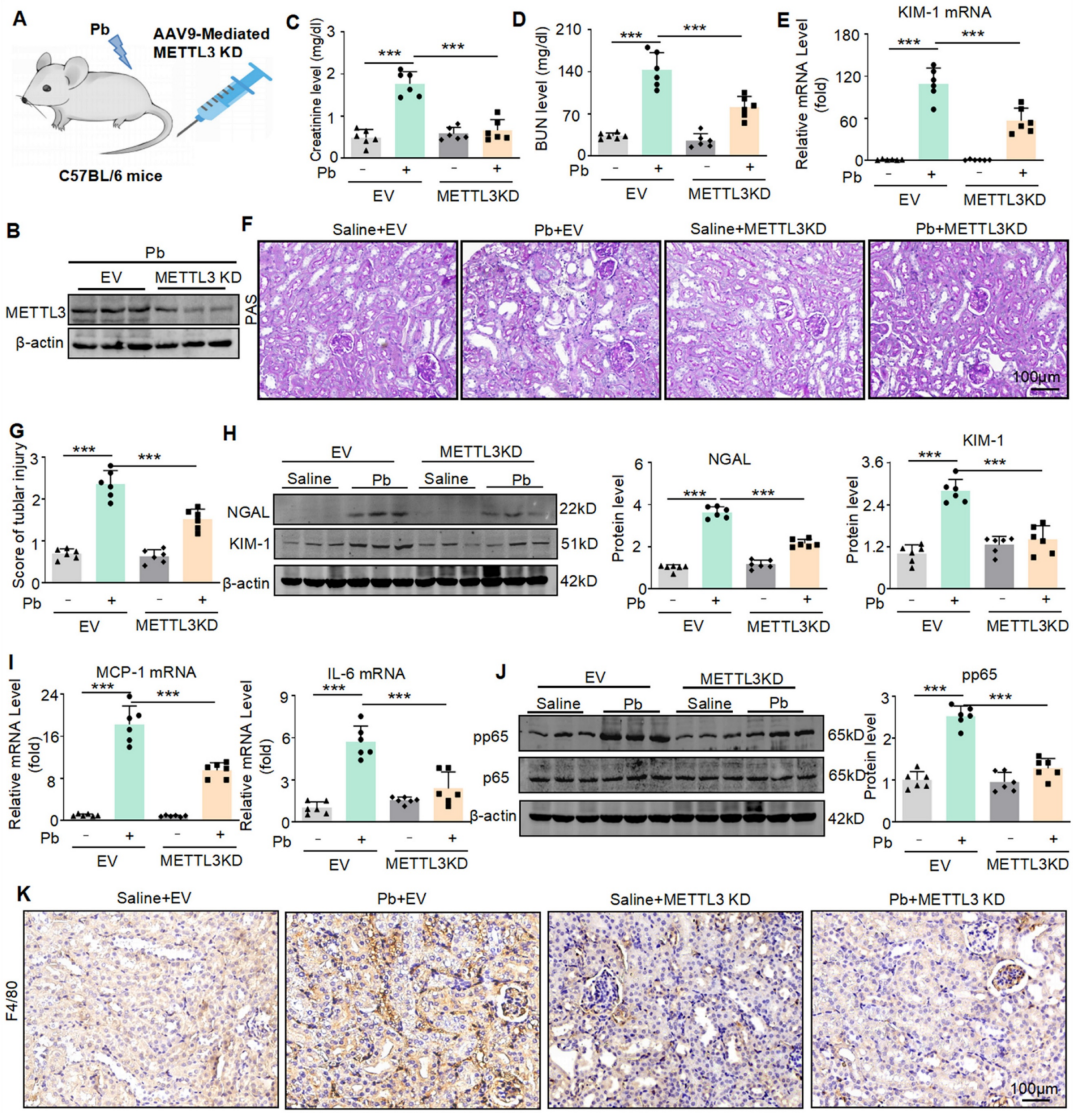
METTL3 knockdown reduces renal injury and inflammatory response in lead acetate-induced mouse. (A) Schematic diagram of AAV9 mediated METTL3 silencing by tail vein injection. (B) The expression of METTL3 in mice. (C and D) Serum creatinine and BUN levels in Pb-induced mice with or without METTL3 knockdown (n = 6 biological replicates, one-way ANOVA with Tukey's multiple comparisons test). (E) Real-time PCR analysis of KIM-1 expression in lead acetate nephropathy with and without METTL3 knockdown (n = 6 biological replicates, one-way ANOVA with Tukey's multiple comparisons test). (F and G) PAS staining and scores for lead acetate-induced lead nephropathy in METTL3 knockdown mice. Scale bars = 100 μm. (H) Western blot and quantitative analysis of NGAL and KIM-1 in lead nephropathy with or without METTL3 knockdown. (I) Real-time PCR analysis of MCP-1 and IL-6 mRNA expression in lead acetate-treated METTL3 knockdown mice (n = 6 biological replicates, one-way ANOVA with Tukey's multiple comparisons test).(J) Western blotting and quantitative analysis of p65 NF-κB phosphorylation in lead nephropathy with or without METTL3 knockdown. (K) IHC staining of F4/80+ macrophage infiltration in mice with lead nephropathy with or without METTL3 knockdown. Scale bars = 100 μm. Data represents the mean ± S.E.M. ***P< 0.001.

To determine the therapeutic potential of METTL3 in lead nephropathy, we silenced METTL3 in mice by injecting AAV9 packaged METTL3 knockdown virus into the tail vein (Figure 7A).

Western blot confirmed that AAV9-packaged METTL3 knockdown plasmid reduced the expression of METTL3 (Figure 7B). We found that the konckdown of METTL3 reduced the abnormally elevated serum creatinine and urea nitrogen levels caused by lead acetate (Figure 7C and 7D). Real-time PCR analyses confirmed a decrease in KIM-1 mRNA expression after METTL3 konckdown (Figure 7E). PAS staining confirmed that silencing METTL3 alleviated pathological damage such as renal tubular dilation and brush-like edge disappearance caused by lead acetate (Figure 7F and 7G). The protein levels of renal injury markers (KIM-1 and NGAL) were elevated in lead acetate induced nephropathy, while they were downregulated in mice injected with AAV9 packaged METTL3 knockout virus. (Figure 7H). In addition, we found that lead acetate increased the expression of pro-inflammatory cytokines (IL-6 and MCP-1), and knockdown of METTL3 reduced the expression of inflammatory cytokines (Figure 7I). Western blot and immunohistochemistry showed that knockdown of METTL3 inhibited activation of p65 NF-κB and reduced the infiltration of F4/80+macrophages (Figure 7J and Figure 7K). In summary, these results indicated that targeting METTL3 was an important approach for treating lead nephropathy.

### The METTL3 inhibitor STM2457 exhibits reno-protective potential *in vitro* and *in vivo*

**Figure 8:**
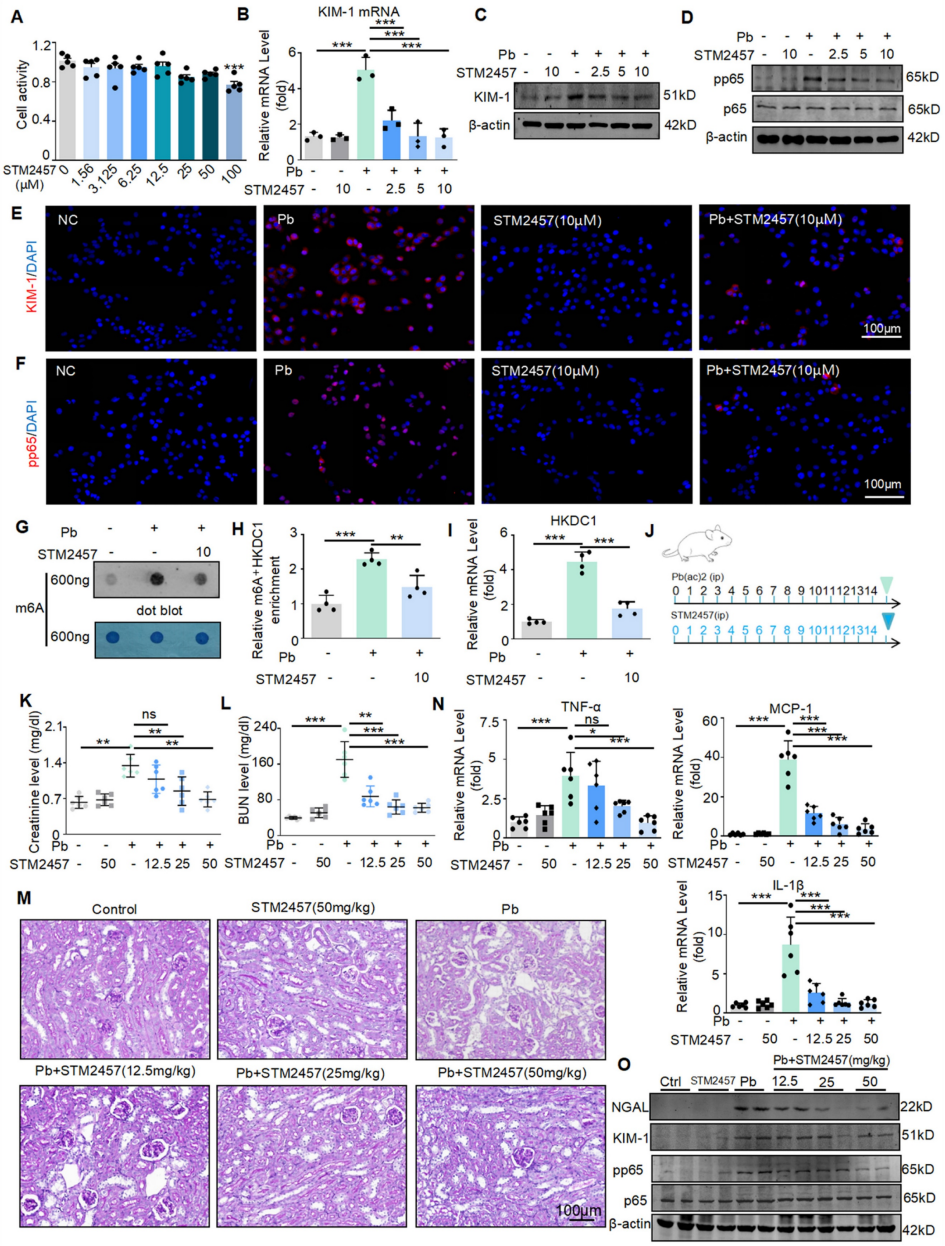
The METTL3 inhibitor STM2457 prevents renal injury and inflammation induced by lead acetate* in vitro* and *in vivo.* (A) Effect of different concentrations of STM2457 on the viability of HK2 cells by MTT assay. (B) Real-time PCR analysis of KIM-1 in STM24574-treated HK2 cells in response to lead acetate treatment (n = 3 biological replicates, one-way ANOVA with Tukey's multiple comparisons test). (C) Western blot analysis of KIM-1 in STM24574-treated HK2 cells in response to lead acetate treatment. (D) Western blot analysis of p65 NF-κB phosphorylation. in STM24574-treated HK2 cells following lead acetate treatment. (E) Representative immunofluorescence staining of KIM-1 in lead acetate-treated HK2 cells with and without STM24574. Scale bars = 100 μm. (F) Representative immunofluorescence staining of pp65 in lead acetate-treated HK2 cells with and without STM24574. Scale bars = 100 μm. (G) Dot blot assay of m6A methylation in lead acetate-treated HK2 cells with and without STM24574. (H) MeRIP-qPCR analysis of alterations in m6A modifications of HKDC1 in lead acetate-stimulated HK2 cells with or without STM24574 (n = 4 biological replicates, one-way ANOVA with Tukey's multiple comparisons test). (I) Real-time PCR analysis of HKDC1 expression in lead acetate-treated HK2 cells with and without STM24574 (n = 4 biological replicates, one-way ANOVA with Tukey's multiple comparisons test). (J) Schematic diagram of STM2457 administration. (K and L) Serum creatinine and BUN levels in lead acetate-induced lead nephropathy mouse models with and without STM2457 treatment (n = 6 biological replicates, one-way ANOVA with Tukey's multiple comparisons test). (M) PAS staining in a lead acetate-induced lead nephropathy mouse model with and without STM2457 treatment. Scale bars = 100 μm. (N) Real-time PCR analysis of MCP-1, TNF-α and IL-1β mRNA expression in lead acetate-treated mice with STM24574 (n = 6 biological replicates, one-way ANOVA with Tukey's multiple comparisons test). (O) Western blot analysis of KIM-1and NGAL levels, and p65 NF-κB phosphorylation in STM2457-treated mice following lead acetate treatment. Data represents the mean ± S.E.M. *P< 0.05, **P< 0.01, ***P< 0.001.

Based on the above findings, to further transform this research, we will explore whether targeted inhibition of METTL3 can be a potential drug for treating Pb-induced nephropathy. STM2457 is a new METTL3 inhibitor with the highest inhibition efficiency among similar compounds. Some studies have found that STM2457 can treat acute promyelocytic leukaemia; however, this has not been studied in kidney diseases. We applied it to *in vitro* and *in vivo* models of lead-induced nephropathy to investigate its renal protective function. First, we evaluated the effect of STM2457 on HK2 cells. The determination of 3- (4,5-dimethyl-2-thiazolyl) -2,5-diphenyltetrazolium bromide assay showed that STM2457 at concentrations less than 12.5 μM had insignificant cytotoxic effects on HK2 cells (Figure 8A). Real-time PCR, Western blot and immunofluorescence analysis showed that STM2457 treatment inhibited the expression of kidney injury indicator KIM-1 in a concentration dependent manner, reduced the release of inflammatory factors, and inhibited NF-κB driven inflammatory response (Figure 8B-E). In addition, immunofluorescence results showed that STM2457 treatment significantly reduced the expression and abundance of pp65 in HK2 cells stimulated with lead acetate (Figure 8F). To detect the inhibitory effect of STM2457 on METTL3 and its impact on m6A modification levels, we conducted dot blot experiments. We found that the m6A level was significantly increased in HK2 cells after treatment with lead acetate, while STM2457 reduced the m6A modification level (Figure 8G). Additionally, MeRIP-qPCR and Real-time PCR analysis revealed that STM2457 decreased the enrichment of m6A antibodies on HKDC1 mRNA and reduced the mRNA expression of HKDC1 (Figure 8H and 8I). Subsequently, we evaluated the renoprotective effects of STM2457 in lead acetate-induced mouse models. Mice were pretreated with STM2457 (12.5, 25, and 50 mg/kg) 6 h prior to lead acetate injection (Figure 8J). Serum creatinine and urea nitrogen levels reduced following STM2457 administration (Figure 8K and 8L). PAS staining demonstrated that STM2457 treatment attenuated Pb-induced renal injury (Figure 8M). The reno-protective effect was further confirmed by STM2457 treatment inhibited the elevation of pro-inflammatory cytokine (TNF-α, IL-1β and MCP-1) in a dosage-dependent manner (Figure 8N). STM2457 inhibited the protein expression of KIM-1 and the phosphorylation level of P65 induced by lead acetate (Figure 8O). Additionally, STM2457 has no toxicity to important organs at therapeutic doses (Figure S4A). Together, these results showed that STM2457 treatment effectively prevented kidney damage both *in vitro* and *in vivo.*

## Discussion

Pb is a toxic heavy metal, and excessive Pb in the blood can cause serious damage to multiple organs in humans 17. Kidneys are important metabolic and excretory organs in the human body. Pb is mainly deposited in the renal tubules, and Pb poisoning can cause thickening of the basement membrane, tubular atrophy, decreased blood flow, and renal perfusion 18. Patients with lead nephropathy may experience symptoms such as low-molecular-weight proteinuria and elevated blood creatinine levels in the early stages, gradually leading to a decrease in glomerular filtration rate. In later stages, mixed proteinuria and tubular acidosis may occur, ultimately leading to end-stage renal disease 19. Lead nephropathy is often an occupational disease, and its clinical treatment often adopts the same treatment plan as that of other kidney diseases, lacking a targeted treatment. Therefore, elucidating the regulatory mechanisms underlying lead nephropathy and identifying better therapeutic targets are of great value for the prevention and treatment of lead nephropathy.

M6A methylation is the most common and abundant modification of RNA, and its function in various diseases has been gradually elucidated 20-22. Our team has focused on kidney injury and epigenetic modifications. Previous studies have found that m6A modification plays an important role in alcoholic nephropathy, diabetic nephropathy, ischaemic nephropathy, and obstructive nephropathy; Due to epigenetic modifications, gene sequences remain unchanged, and phenotypes vary depending on the environment. In terms of environmental pollution, Pb pollution is the most common and has a significant impact on the kidneys 23; therefore, this study was conducted. In this study, we provided convincing evidence that upregulated METTL3 and overactivated m6A modifications are closely associated with Pb-induced nephropathy. Elevated METTL3 promotes m6A modification of HKDC1 mRNA and inhibits its ubiquitination degradation by binding to ATPB, promoting kidney injury and the inflammatory response. Subsequently, we used the small molecule inhibitor STM2457 of METTL3 and demonstrated that it significantly inhibited the METTL3/HKDC1 m6A axis, thereby preventing kidney injury and inflammation. Collectively, these results revealed that the METTL3/HKDC1/ATPB axis is a potential therapeutic target for renal injury and inflammation.

A lead nephropathy animal model was established by intraperitoneal injection of lead acetate into mice. The kidney was most severely damaged two weeks after injection, and three weeks after injection, which may be due to the kidney being in the stage of injury repair, leading to a slight decrease in blood creatinine and urea nitrogen levels. The accumulation of lead acetate in the kidneys mainly damages renal tubular epithelial cells; therefore, we constructed conditional knockout mice of renal tubular epithelial cells to explore their function. *In vitro* experiments, we used renal tubular epithelial cells as the cell type affected by lead acetate and explored the dose and duration of cell damage caused by lead acetate. We found a significant increase in m6A modification levels in lead nephropathy *in vivo* and *in vitro*, mediated by the methyltransferase METTL3 rather than other transferases. In terms of function, conditional knockout of METTL3 in renal tubular epithelial cells and siRNA silencing in HK2 cells *in vitro* reduced Pb-induced renal injury and inflammation. Consistent with this, overexpression of METTL3 *in vitro* can exacerbate damage, but knockdown of METTL3 does not reduce Pb accumulation in the kidneys. These findings suggest that METTL3 promotes kidney injury and mediates inflammation. Therefore, METTL3 may represent a novel intervention strategy for the treatment of Pb-induced kidney injury and inflammation.

Next, we determined the detailed mechanism by which METTL3 exacerbates Pb-induced nephropathy. We performed transcriptome and m6A methylation sequencing of renal tissues from lead acetate-treated METTL3 mice with conditional knockout of renal tubular epithelial cells and lead acetate-treated wild-type mice. These results indicated that multiple pathways enriched in m6A methylation are directly related to inflammatory regulation, which is consistent with our findings. Cross-analysis was performed between genes with significantly downregulated m6A methylation and those with significantly altered mRNA levels. Multiple experimental methods were used to screen and identify HKDC1 as the optimal target for METTL3 regulation. Western blot validation results indicated that METTL3 silencing reduced the expression level of HKDC1, whereas METTL3 overexpression increased the expression level of HKDC1. Hexokinases are the first rate-limiting enzymes in glycolysis and include five subtypes: HK1, HK2, HK3, HK4, and Hexokinase domain protein 1 (HKDC1, HK5) 24. HKDC1 is a recently discovered enzyme that acts independent of other enzymes. To date, researchers have gained some understanding of the role and mechanism of HKDC1 in tumours and inflammatory diseases 25,26; however, its role in the kidneys has not yet been reported. We found that HKDC1 was also expressed in renal tubular epithelial cells and was significantly upregulated in lead nephropathy mice and lead acetate stimulated HK2 cells. More importantly, silencing HKDC1 with AAV9 *in vivo* and siRNA can alleviate lead induced kidney injury and inflammation, as well as the activation of inflammatory transcription factor NF-κB in renal tubular epithelial cells. A recent report showed that the mitochondrial localisation of HKDC1 plays a crucial role in liver cancer progression by regulating ATP levels 27; however, the relationship between HKDC1 and ATP is currently unclear. Previous studies have also found that HKDC1 is localised in the mitochondria and binds to mitochondrial proteins, such as PHB2, to maintain mitochondrial function. 26, 28 To further explore the role of HKDC1 in the kidney, we used mass spectrometry to identify proteins that bind to HKDC1. From the sequencing results, we found that ATPB is located in the mitochondria and is produced by ATP. We found that ATP and ATPB were also elevated in a Pb-induced kidney disease model, and ATPB bound to HKDC1 in the mitochondria. To further investigate the binding site of HKDC1 on ATPB and how it promotes its expression, we constructed different truncated forms of ATPB, and co-immunoprecipitation experiments confirmed that HKDC1 binds to ATPB. The binding of HKDC1 to ATPB antagonises the ubiquitination enzyme Murf1 and its binding site, thereby inhibiting ubiquitination and increasing its expression. Inflammatory response is a common pathogenic feature of various kidney diseases 29. High expression of ATPB and synthesis of high ATP levels also promote NF-κB activation and inflammatory response 30.

It is worth noting that the fate of the target transcript is usually correlated with m6A readers, and the modified reading proteins that mediate m6A include YTH domain family proteins (YTHDF1,2,3), insulin-like growth factor 2 mRNA binding proteins (IGF2BPs), and heterogeneous ribonucleoprotein (HNRNPs) family proteins 31,32. At present, a class of proteins containing the YTH functional domain has been identified as m6A modified binding proteins. YTHDF1-3 and YTHDC1-2 have been confirmed to be binding proteins m6A YTHDF1 mainly affects the translation of m6A modified genes 33, YTHDF2 mainly affects the degradation of m6A modified genes 34, and YTHDC1 binding to m6A modified genes affects their splicing 35. HNRNPC is a rich nuclear RNA binding protein involved in pre mRNA processing, and studies have shown that HNRNPC regulates the abundance and selective splicing of target transcripts through m6A RNA binding 36. The IGF2BP family members, including IGF2BP1, IGF2BP2, and IGF2BP3 common m6A readers, recognize m6A modifications in thousands of messenger RNA transcripts, promote the stability and translation of these messenger RNAs, and thus affect the outcome of normal gene expression and stress conditions 37. For example, IGF2BP2 mediated MIS12 increases the stability of messenger RNA through METTL3 mediated m6A modification, thereby reversing the phenotype of aging human mesenchymal stem cells 38. In this study, we confirmed that IGF2BP2, rather than IGF2BP1/3, plays a crucial role in the stability of HKDC1 mRNA under the control of METTL3.

We evaluated the efficacy of METTL3 targeted therapy using the reported small molecule inhibitor STM2457 39. A recent study found that STM2457 exhibited therapeutic effects in myeloid leukaemia, indicating that METTL3 targeted therapy may be effective against this disease. Unfortunately, the exploration of METTL3 inhibitors is still at an early stage. STM2457 was more effective in restoring renal dysfunction and inhibiting renal injury and inflammation. Therefore, STM2457 is a promising METTL3 inhibitor that can be used in further studies, and has potential clinical applications.

In summary, our study suggests that METTL3 mediates the m6A methylation of HKDC1 and binds to ATPB to promote renal injury and inflammatory responses. The METTL3/HKDC1/ATPB axis is a potential therapeutic target for Pb nephropathy. In addition, STM2457 may act as a protective agent against lead acetate-induced kidney injury.

## Supplementary Material

Supplementary figures and tables.
