# Supplementary material for: METTL3 mediated m6A modification of HKDC1 promotes renal injury and inflammation in lead nephropathy
Source: Int J Biol Sci. 2025 May 31;21(8):3755–75. doi: 10.7150/ijbs.112463 (PMC12160926; doi:10.7150/ijbs.112463)
Supplement: Supplementary file 1 — Supplementary figures and tables. [file ijbsv21p3755s1.pdf]

Supplementary figures

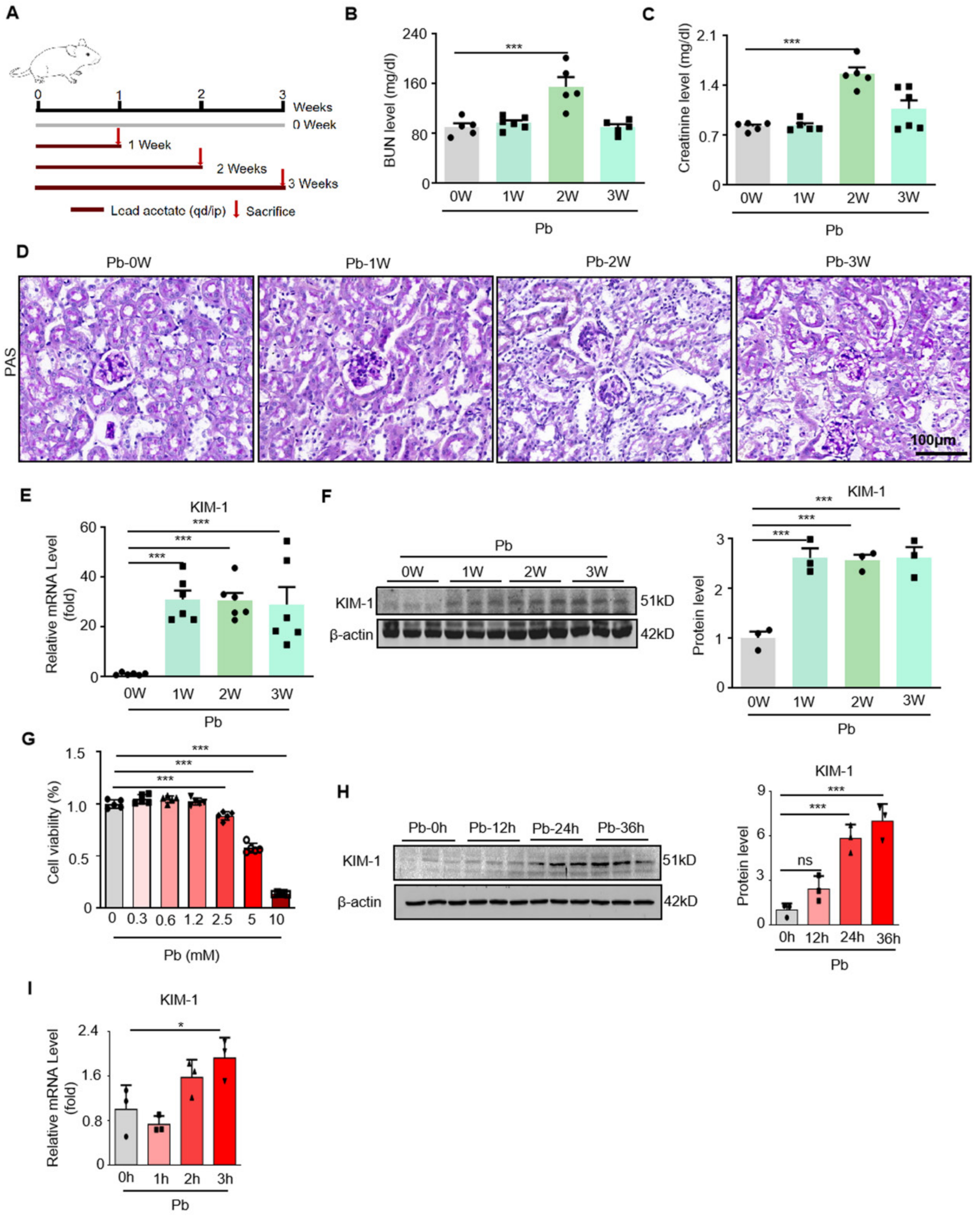

Figure S1. Construction of the lead nephropathy models *in vitro* and *in vivo*. (A) Schematic diagram of *in vivo* model. (B) Serum BUN level in lead acetate-induced lead nephropathy mouse models. (C) Serum creatinine level in lead acetate-induced lead nephropathy mouse models. (D) Periodic acid-Schiff (PAS) staining in a lead acetate-induced lead nephropathy mouse model. (E) Real-time PCR analysis of KIM-1 mRNA expression in lead acetate-treated mice. (F) Western blot and

quantitative analysis of KIM-1 level in lead nephropathy mouse model. (G) Effect of different concentrations of Pb on the viability of HK2 cells by MTT assay. (H) Western blot and quantitative analysis of KIM-1 level in lead acetate-treated HK2 cells. (I) Real-time PCR analysis of KIM-1 mRNA expression in lead acetate-treated HK2 cells. Data represents the mean ± S.E.M. **P*< 0.05, ****P*< 0.001.

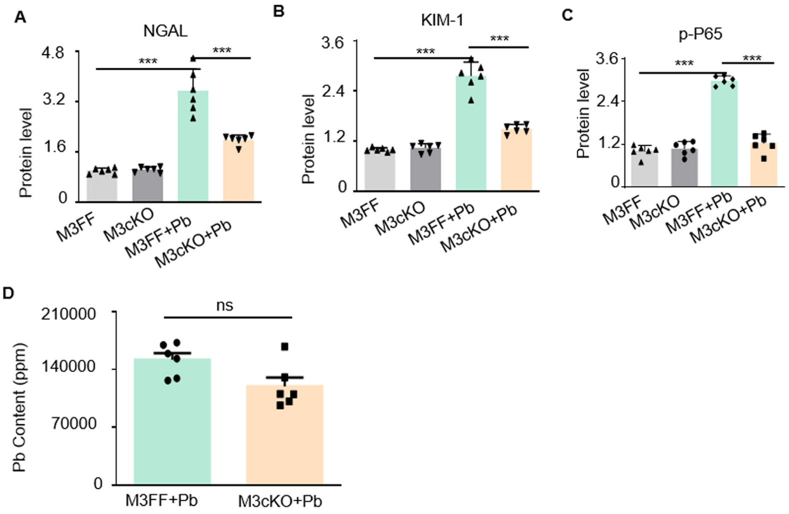

Figure S2. The effect of METTL3 cKO on renal injury protein and lead content induced by lead acetate. (A) Quantitative analysis of NGAL level in METTL3$^{Flox/Flox}$ and METTL3 cKO mice exposed to Pb. (B) Quantitative analysis of KIM-1 level in METTL3$^{Flox/Flox}$ and METTL3 cKO mice exposed to Pb. (C) Quantitative analysis of pp65 level in METTL3$^{Flox/Flox}$ and METTL3 cKO mice exposed to Pb. (D) Quantitative analysis of Pb content in METTL3$^{Flox/Flox}$ and METTL3 cKO mice exposed to Pb. Data represents the mean ± S.E.M. ****P*< 0.001.

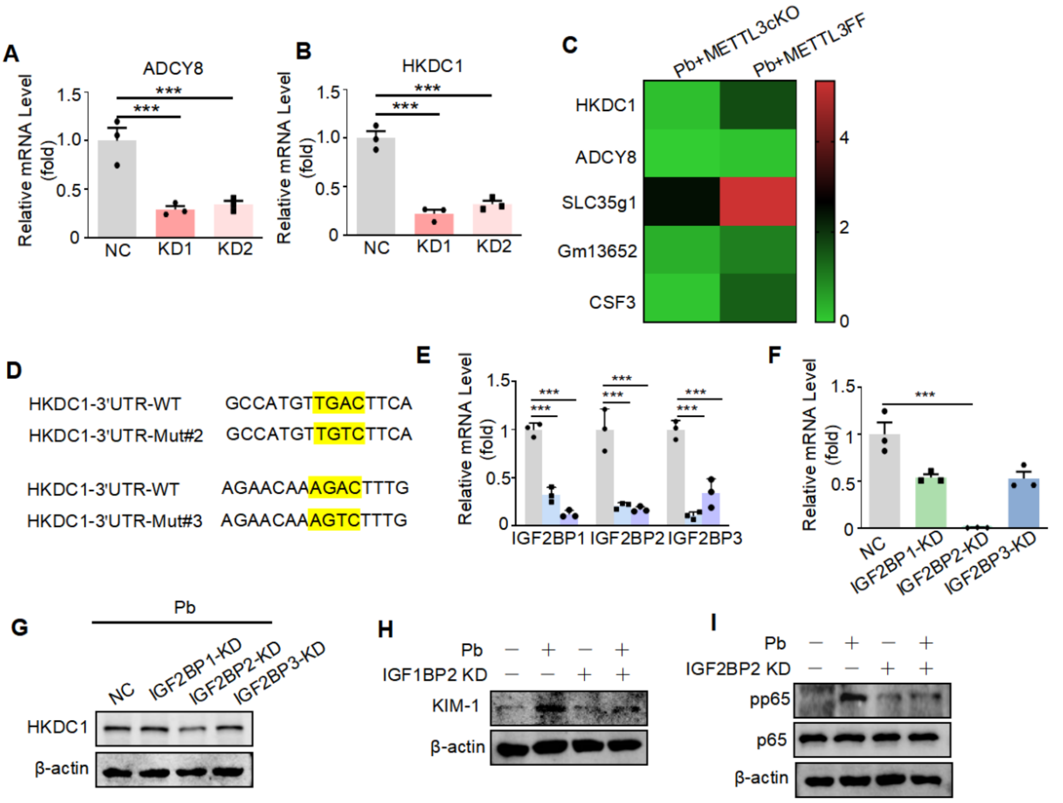

Figure S3. IGF2BP2 stabilizes METTL3 regulation of HKDC1 m6A modification. (A and B) Knockdown of ADCY8 and HKDC1 was confirmed using Real-time PCR. (C) Heat map analysis of changes in methylation levels by m6A-MeRIP-Seq. (D) Modification mutation site of m6A methylation in HKDC1. (E)Validation of IGF2BP1, IGF2BP2 and IGF2BP3siRNA silencing efficiency. (F)The effect of silencing IGF2BP1, IGF2BP2 and IGF2BP3 on HKDC1 mRNA. (G) The effect of silencing IGF2BP1, IGF2BP2 and IGF2BP3 on HKDC1 protein. (H) Western blot analysis of KIM-1 in lead acetate-treated HK2 cells with and without HKDC1 knockdown. (I) Western blot analysis of pp65 and p65 in lead acetate-treated HK2 cells with and without HKDC1 knockdown. Data represents the mean ± S.E.M. ****P*< 0.001.

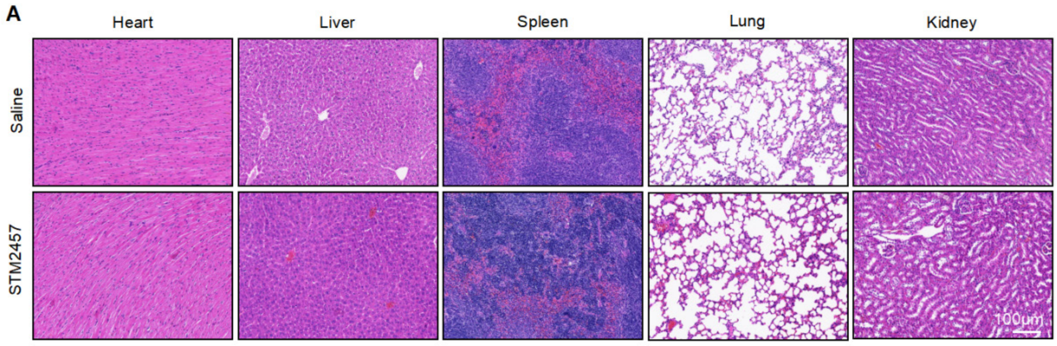

Figure S4. The effect of STM2457 on heart, liver, spleen, lungs, and kidneys in mice. (A) HE stainingof important organs with or without STM2457 in mice. Scale bars = 100 μm.

Table S1. Primer sequences used in Real-time PCR

Genes(Mouse)	Forward primer (5'-3')	Reverse primer (5'-3')
METTL3	CCCAACCTTCCGTAGTGATAG	TGGCGTAGAGATGGCAAGAC
METTL14	GGTCGGAGTGTGAACCTGAT	GGTCCTCTTCCACGCTGTAT
WTAP	TAATGGCGAAGTGTCGAATG	CTGCTGTCGTGTCTCCTTCA
FTO	TCACAGACGTGGTTTCCGAG	ACCACTGGGTTGAGAGGAGT
ALKBH5	TGCTGCGTATGGGGCTTAAA	ATGCCTAACAGGAGCAACCC
KIM-1	CAGGGAAGCCGCAGAAAA	GAGACACGGAAGGCAACCAC
TNF-α	CATCTTCTCAAAATTCGAGTGACAA	TGGGAGTAGACAAGGTACAACCC
IL-6	GAGGATACCACTCCCAACAGACC	AAGTGCATCATCGTTGTTCATACA
IL-1β	GCTTCAGGCAGGCAGTAT	ACAAACCGCTTTTCCATCT
MCP-1	CTTCTGGGCCTGCTGTTCA	CCAGCCTACTCATTGGGATCA
HKDC1	ACACTTGGTGGCGTTTTACTT	CCGCATGTGATACAGGAACC
β-actin	CATTGCTGACAGGATGCAGAA	ATGGTGCTAGGAGCCAGAGC

Table S2. Primer sequences used in Real-time PCR

Genes(Human)	Forward primer (5'-3')	Reverse primer (5'-3')
METTL3	GCCTTTGCCAGTTCGTTAGT	TGACCTTCTTGCTCTGTTGTTC
METTL14	AAATGCTGGACTTGGGATGA	TGAGGCAGTGTTCCTTTGTTC
WTAP	TGCCCAACTGAGATCAACAA	CATTCGACACTTCGCCATTA
FTO	GGTTGATAAGGCACAAGGCA	TCAGCAGGTAATGTTCGGGC
ALKBH5	GCAGAGTTGTTCAGGTTGCC	GTCAGGACCACTGCACTAGC
KIM-1	CTGCAGGGAGCAATAAGGAG	TCCAAAGGCCATCTGAAGAC
TNF-α	CCCAGGGACCTCTCTCTAATCA	GCTACAGGCTTGTCACTCGG
IL-6	CGGGAACGAAAGAGAAGCTCTA	GAGCAG CCCCAGGGAGAA
IL-1β	ACTACAGCAAGGGCTTCAGG	CATATCCTGTCCCTGGAGGT
MCP-1	AGCAGCAAGTGTCCCAAAGA	GGTGGTCCATGGAATCCTGA
HKDC1	GTTGCCCACCTTCGTCAGG	AGCGACTTGCACCTTCAGC
IGF2BP1	GCGGCCAGTTCTTGGTCAA	TTGGGCACCGAATGTTCAATC
IGF2BP2	TGGAAGCGCATATCAGAGTG	AGCAAAGAAGTGCCCGATAA
IGF2BP3	TATATCGGAAACCTCAGCGAGA	GGACCGAGTGCTCAACTTCT
ADCY8	CCTGCGGCACCAAAGTCTT	CGAGTTGCTAGGGGCACAG
CSF3	GCTGCTTGAGCCAACTCCATA	GAACGCGGTACGACACCTC
β-actin	CGCCGCCAGCTCACCATG	CACGATGGAGGGGAAGACGG
